# Supplementary material for: The use of carbogen for interruption of febrile seizures - the randomized controlled CARDIF trial
Source: PLoS One. 2025 Dec 23;20(12):e0324422. doi: 10.1371/journal.pone.0324422 (PMC12725555; doi:10.1371/journal.pone.0324422)
Supplement: S1 File — (DOCX) [file pone.0324422.s001.docx]

**Study protocol for the clinical trial:**

**CARbon DIoxide in the treatment of febrile convulsions (CARDIF)**

A monocentric, prospective, double-blind, randomized, *placebo*-controlled study

**Eudra-CT Number: 2011-001403-12 Version: 1.2 from 01.03.2012**

**Test plan code: CARDIF**

**Sponsor: Charité - Universitätsmedizin Berlin**Charitéplatz 1, 10117 Berlin

**Representative of the sponsor: Prof. Dr. med. Markus Schülke-Gerstenfeld**
Charité - Universitätsmedizin Berlin
Clinic for Neuropediatrics
Augustenburger Platz 1
13353 Berlin
and
NeuroCure Clinical Research Center NCRC
Charité Campus Mitte, Charitéplatz 1, 10117 Berlin
Tel.: 030 450 566 468, Fax 030 450 566 920

**Principal investigator:** **Dr. med. Ulrike Grieben**
Experimental and Clinical Research Center
Lindenberger Weg 80, 13125 Berlin
Phone: 030 450 540 507, Fax: 030 450 540 907

**Biometrics: Prof. Dr. rer. nat. Peter Martus**Charité - Universitätsmedizin Berlin
Institute of Biometry and Clinical Epidemiology
Charité Campus Mitte, Charitéplatz 1, 10117 Berlin
Phone: 030 450 562 162, Fax: 030 450 562 972

________________________________________________________________________

The following persons agree to the contents of this protocol by signing it and confirm that they are aware of the ICH GCP guidelines, the requirements of the AMG and the GCP regulation and that the clinical trial will be conducted in accordance with these regulations.

#

**Berlin, 01.03.2012**

**Sponsor representative: Prof. Dr. med. Markus Schülke-Gerstenfeld**

**Berlin, 01.03.2012**

**Principal investigator: Dr. med. Ulrike Grieben**

**Table of contents**

[**1**](#_rqdm886jq6cf) **Synopsis 4**

[**2**](#_y3vih9cvvg2v) **Introduction 8**

[2.1](#_imwb9wq4t59) Background 8

[2.2](#_siudzfx2c75i) State of knowledge about the investigational medication 10

[*2.2.1*](#_5az9y996kffg) *Substance name, manufacturer, approval status, chemical and physical properties 10*

[*2.2.2*](#_g6xn0onvccnp) *Side effects, tolerability, effects on physiological parameters 11*

[*2.2.3*](#_1hjec4de89of) *Pharmacokinetics 11*

[*2.2.4*](#_uomff224f8gw) *Mechanism of action 11*

[*2.2.5*](#_i29qzgjcyf1) *Results of clinical trials conducted to date 12*

[*2.2.6*](#_a0421gcuscjk) *Question and justification of the project (rationale) 12*

[**3**](#_976luuw6z56u) **Aims of the clinical trial 13**

[3.1](#_j2hnnor5v0b8) Primary endpoints 13

[3.2](#_mlmkj92lvg0h) Secondary endpoints 13

[3.3](#_fecsyx656e8o) Study design 13

[3.4](#_77yhmsv3yxr6) Schedule 14

[**4**](#_9j9ynnnlahwm) **Selection of patients 14**

[4.1](#_85z7qmyu26ov) Inclusion criteria 14

[4.2](#_x9a8dkhrq3ij) Exclusion criteria 14

[**5**](#_3f3shat9l58j) **Treatment plan and study procedure 15**

[5.1](#_8gg9lixsks3q) Recruitment, screening and inclusion procedures 15

[5.2](#_mcvwugsohu1f) Clinical examinations and ward round schedule 15

[*5.2.1*](#_chkzzk9byaro) *Description of the measures and deviation from the routine 16*

[5.3](#_8w7o3t8e48jt) Description of the test medication 16

[*5.3.1*](#_xwv90ahqyk0t) *List of side effects and interactions 17*

[*5.3.2*](#_4gyhkeoyrk4w) *Treatment scheme 17*

[*5.3.3*](#_9z2nocyqc2sj) *Storage, issue and return 18*

[5.4](#_c1y54kjsgdsh) Concomitant medication / concomitant therapy / rescue medication 18

[5.5](#_5c85wavs9dcp) Blinding, emergency envelopes, unblinding 18

[**6**](#_l6dduiqyv6rl) **Risk-benefit assessment 18**

[**7**](#_h2kej1ncz9y6) **Termination and further treatment 20**

[7.1](#_j7i819izb8cz) Premature discontinuation of a single patient 20

[7.2](#_42iyj0b8rr3n) Premature termination of the entire clinical trial 21

[7.3](#_gvi3m3xly8yy) Plan for further treatment after graduation/completion 21

[**8**](#_53yh1qrp4tje) **Pharmacovigilance 21**

[8.1](#_3ihm3swmkjvz) Definitions 21

[8.2](#_xi48bqdaapyx) Assessment of intensity 22

[8.3](#_g3m5gqva45rx) Assessment of the causal relationship 22

[8.4](#_jyz9onpf5pr1) Documentation of AEs and SAEs 22

[8.5](#_ta6o1zhvftri) Notification of SAEs by the auditor 23

[8.6](#_z2cagxfhwqgq) Notification of (S)AEs and SUSARs by the sponsor 23

[8.7](#_aqwnashlmdgp) Expected adverse events 24

[8.8](#_sj74ywv4rl49) Exception to the SAE definition 24

[8.9](#_ybcflgiuh0yh) Documentation, data storage and access to data 24

[**9**](#_lhf7n17xg9m4) **Quality management 25**

[**10**](#_chiab5z7632t) **Statistical analysis 25**

[10.1](#_jgvb10b133me) Study design 25

[10.2](#_yeyxllz4iy02) Target variables (endpoints) 26

[10.3](#_kp1b8xigx5q2) Hypotheses 26

[10.4](#_pw0z6flcmqcp) Statistical evaluation methods 26

[10.5](#_k44tabvldl0) Determination of the optimal sample size (number of cases) 26

[10.6](#_jncu24grc24z) Interim evaluations 27

[10.7](#_ujpid1q2tzud) Definition of evaluation collectives 27

[10.8](#_ilwav9f3c0hm) Randomization, stratification and blinding 27

[10.9](#_z74g1el3mpr) Statistical data processing and biostatistical support of the study 27

[10.10](#_3vzn7v8fsht) Documentation 27

[**11**](#_umzrxmi0exh9) **Ethical, legal and administrative aspects 27**

[11.1](#_28cw8nrwbhqx) Legal aspects 27

[11.2](#_xrxv7m89uujz) Permits 28

[11.3](#_tl7vg3215zv4) Patient information and consent 28

[11.4](#_uqh2x0b9qt25) Test person insurance 29

[11.5](#_md1f5dyab95d) Data protection 29

[11.6](#_1qiu4jbisqhk) Publication of the results of the study 29

[**12**](#_jxp4uoj1rqzu) **Bibliography 29**

[**13**](#_m6adxb7lwbj6) **Attachments 32**

# Synopsis

| **Title of the study** | **Carbon Dioxide in the treatment of febrile convulsions  (CARDIF)** |
| --- | --- |
| **Sponsor** | Charité - Universitätsmedizin Berlin |
| **Representative of the sponsor** | **Prof. Dr. med. Markus Schülke-Gerstenfeld** Charité - Universitätsmedizin Berlin Clinic for Neuropediatrics Augustenburger Platz 1, 13353 Berlin  and  NeuroCure Clinical Research Center (NCRC) Charité Campus Mitte, Charitéplatz 1, 10117 Berlin Tel.: 030-450 566468, Fax 030-450 566920 |
| **Principal investigator** | **Dr. med. Ulrike Grieben** Experimental and Clinical Research Center Lindenberger Weg 80, 13125 Berlin Phone: 030 450 540 507, Fax: 030 450 540 907 |
| **Test center** | **Charité - Universitätsmedizin Berlin with the locations:**  Clinic for Neuropediatrics, Campus Virchow Klinikum, Augustenburger Platz 1, 13353 Berlin (CVK)  and  NeuroCure Clinical Research Center NCRC (CCM), Charité Campus Mitte, Charitéplatz 1, 10117 Berlin  and  Experimental and Clinical Research Center, Lindenberger Weg 80, 13125 Berlin (CBB) |
| **Biometrics** | **Prof. Dr. rer. nat. Peter Martus** Charité - Universitätsmedizin Berlin Institute of Biometry and Clinical Epidemiology  Charité Campus Mitte, Charitéplatz 1, 10117 Berlin Tel. : 030 450 562 162, FAX: 030 450 562 972 |
| **Accompanying scientific program** | No accompanying scientific program is planned. |
| **Study design** | prospective, monocentric, double-blind, randomized, *placebo*-controlled; two-phase adaptive design according to Bauer & Köhne (BAUER *et al.*, 1994) |
| **Hypothesis** | Carbogen gas (5% CO_2_, 95% O_2_) is effective in interrupting acute febrile convulsions and is therefore superior to *placebo* (100% O_2_) |
| **Question based on scientific background** | 5% of all children suffer at least one febrile convulsion in their lifetime. 30% of these children have repeated or particularly long-lasting febrile convulsions, which is always a very threatening situation for parents. The current standard therapy is the rectal administration of diazepam. This substance has a sedative effect, but unfortunately does not always work and the children usually sleep for the rest of the day or are at least drowsy.  In an animal experiment conducted by Schuchmann *et al.* on young rats, they were able to show that the inhalation of 5% carbon dioxide (CO_2_) led to the immediate interruption of the seizure in the rat model (Schuchmann *et al.* Experimental febrile seizures are precipitated by a hyperthermia-induced respiratory alkalosis. *Nature Med* **12**, 817-823, 2006).  These findings have now prompted us to test this rapidly effective and side-effect-free therapy in animal experiments in a clinical study on humans. The data to date comprise both animal experiments and clinical descriptions in which rebreathing led to an increase in the CO_2_ content of the blood and rapid cessation of seizures:  [1] Tolner *et al.* Suppression of cortical epileptic activity in rats and humans by carbon dioxide. *Epilepsia* **50 (Suppl)**, 98, 2009  [2] Schuchmann *et al.* Suppression of complex febrile seizures by elevating respiratory CO_2_ using a rebreathing technique - two case reports. *Epilepsia* **50 (Suppl),** 245, 2008  However, such rebreathing of exhaled air outside a clinic - seizures usually take place at home - is impractical or potentially even dangerous without the necessary monitoring measures by parents, as it can lead to an excessive increase in CO_2_ partial pressure in the blood.  A safe alternative is the use of carbogen gas (95% oxygen + 5% carbon dioxide). The high oxygen content ensures that no asphyxiation can occur and the carbon dioxide leads to the desired acidification of the blood, which then suppresses the epileptic discharges in the brain.  The following publication describes such an application of carbogen gas for the successful suppression of electroencephalographically detected seizures in 7 patients in a controlled clinical setting:  [3] Tolner *et al.* Five percent CO₂ is a potent, fast-acting inhalation anticonvulsant. *Epilepsia* **52**, 104-14, 2011  Based on the above-mentioned preliminary findings, we would now like to conduct a clinical study to test the effectiveness of carbogen gas for interrupting acute febrile convulsions. If this therapy proves to be effective and safe, the administration of diazepam, which has many side effects, could be replaced in future. |
| **Investigational medication** | **Test medication:** Carbogen gas in a low-pressure gas can **Dose**: Single dose of 6 liters over 3 minutes during a seizure.  **Route of administration:** Administration via a breathing mask |
| **Comparative medication** | ***Placebo*:** 100% oxygen (O_2_) |
| **Schedule** | **Start of recruitment after approval:** QIII/2011 **Duration of recruitment:** 24 months **Duration of treatment:** up to 3 minutes per event (acute treatment)  **Duration of study per participant:** 24 months |
| **Total number of patients** | A total of 288 children will be recruited, 80 for the first and 208 for the second study phase. Seizures are expected in 25% of these children, so that a total of 20 + 52 = 72 children are included in the analysis in the sense of a "modified ITT population" (EMA, CPMP/EWP/558/95 rev2). The number can be corrected upwards, but not downwards, after completion of the first study phase. |
| **Study population** | Patients with at least one febrile convulsion aged between 12 months and 5 years; infants aged 6 months and older can also be included after a positive result of the interim safety analysis according to Bauer & Köhne. |
| **Inclusion criteria (selection)** | **Diagnosis:** condition after at least one febrile convulsion **Age from** 12 months **to** 5 years until the interim analysis, after a positive result of the interim safety analysis according to Bauer & Köhne, infants from 6 months can also be included  **Gender:** patients of both sexes |
| **Exclusion criteria (selection)** | [1] Serious organ disease  [2] Underlying neurological disease  [3] Pulmonary disease (e.g. asthma)  [4] Detection of hypersynchronous activity in the interval EEG  [5] Cerebral seizures without fever  [6] Known incompatibility of Carbogen  [7] Insufficient ability of the legal guardians to communicate (e.g. due to lack of language skills) |
| **Documentation times** | [1] Clarification / Inclusion*  [2] Randomization*  [3] Outpatient presentation after each seizure  [4] Telephone visits: every 6 months  * can take place on the same day |
| **Endpoints** | **The primary endpoint of the study is:**  **[1]** The efficacy of carbogen inhalation determined by the number of patients with acute febrile convulsion in whom the test medication was used but was unsuccessful within the administration time interval of 3 minutes and standard therapy (rectal diazepam administration) was initiated. (Comparison of the *verum* group with the *placebo* group)  **Secondary endpoints are:**  **[1]** The safety of the application of 6 liters of carbogen *via* a low pressure cylinder with a breathing mask to interrupt acute fiber convulsions in the home situation by parents.  **[2]** Manageability of Carbogen low-pressure doses with attached breathing mask for interrupting febrile convulsions at home and on the move (mobility)  **[3]** Quality of life  **[4]** Satisfaction and anxiety of parents |
| **Security** | After each febrile convulsion, the parents are called to the study center to assess the outcome of the acute treatment and to record possible adverse events and side effects. |
| **Termination criteria (selection)** | **In individual cases:**  **[1]** Withdrawal of the consent of the legal guardians  **[2]** Subsequent occurrence of an exclusion criterion  **[3]** Frequent SAEs for which the causal relationship to the study medication cannot be excluded.  **[4]** Unauthorized use of the study drug or non-compliance of the guardians (defined as at least two febrile seizures without timely use of the study medication)  **Termination of the overall study:**  **[1]** More than 30% single-case drop-outs |
| **Statistical evaluation** | The evaluation is carried out according to Bauer Köhne (1994) with〈 =0.025, c_〈_ =0.00380 and〈 _0_=0.5 (one-sided in each case). The main outcome variable diazepam administration no (success)/yes (failure) is evaluated as a binary endpoint using exact Fisher tests. Assuming a difference of 75% *versus* 25% for 2*26 patients, the power for the two-sided Fisher test is 86% (worst case p_1_=c /_〈_〈 _0_). The secondary endpoint "manageability" is evaluated by relative frequency + two-sided 95% confidence interval. |
| **Possible risks, side effects, contraindications,** | **Possible side effects of Carbogen:** The desired increase in pCO_2_ in the blood to a maximum of 5% (corresponding to 37.5 mmHg; normal range 30-40 mmHg) is still within the physiological range and could possibly lead to increased respiratory drive and the short-term subjective feeling of breathlessness (similar to holding one's breath). Patients with an underlying pulmonary disease are therefore excluded from the study.  The brief administration of 95% or 100% oxygen over 3 minutes is very unlikely to lead to oxygen toxicity (PUCCIO *et al.*, 2009) .  Apart from the administration of carbogen/*placebo*, there are no study-related measures. |
| **Risk-benefit assessment** | The overall risk of the study is considered to be very low. The application of the carbogen doses described above does not pose any risks because the carbogen administration only restores a physiological state.  The patient is not deprived of any standard therapy. If the use of the study medication does not end the seizure, the standard treatment (rectal administration of 5 mg diazepam) is used after 3 minutes, in accordance with the guidelines (CAPOVILLA *et al.*, 2009) . This means that the low-risk study intervention is only used in the time window in which, according to the guidelines, no therapy would take place.  Each individual seizure event is evaluated and documented by a member of the project team and the child is promptly seen and clinically examined in the study outpatient clinic. If the parents are unable to come to the study outpatient clinic, we offer the parents a home visit by a medical colleague. No study-related blood samples will be taken. |

# Introduction

## Background

**Epidemiology and clinic:** Febrile seizures are the most common form of cerebral seizures in children. Depending on ethnicity, these seizures occur in 3-7% of the population (NELSON *et al.*, 1976) , and in 23-42% of cases (depending on the study) also occur repeatedly. (KNUDSEN 2000) Febrile convulsions occur solely due to increased body temperature, typically only occur between the ages of 6 months and 5 years, have a good prognosis and are not associated with cerebral damage. Seizures that occur before or after this critical age range must be investigated in particular to determine whether there is another pathogenesis (e.g. inflammation of the brain substance, brain malformations or cerebral hemorrhage). The occurrence of febrile convulsions is associated with an increased risk of 2-8% of developing epilepsy later in life, compared to 1% in the normal population (ANNEGERS *et al.*, 1987; BERG 1992). There is no evidence that drug treatment of febrile convulsions prevents the later occurrence of epilepsy (BAUMANN *et al.*, 2000). There is also no evidence that timely reduction of fever prevents the occurrence of febrile convulsions (KNUDSEN 2000; SCHNAIDERMAN *et al.*, 1993).

**Risk factors for febrile convulsions:** Risk factors for the occurrence of febrile convulsions and recurrent febrile convulsions are (1) a positive family history of cerebral seizures and febrile convulsions, (2) young age at first manifestation, (3) low temperature and (4) shorter duration of fever at the onset of cerebral seizures, (5) frequent febrile infections (BERG *et al.*, 1997; OFFRINGA *et al.*, 1994). The following recurrence risks result: 10% without risk factors; 25-50% 1-2 risk factor(s); 50-100% 3 or more risk factors (KNUDSEN 2000). "Simple febrile convulsions" often occur within the first 24 hours after the onset of fever and only occur once during a fever period. Complicated febrile convulsions" occur if they last longer than 15 minutes, affect only one side of the body or occur several times during a fever episode.

**Current anticonvulsant drug therapy with a benzodiazepine:** Most febrile convulsions last less than 5 minutes and are usually self-limiting. In recurrent febrile convulsions, the seizure duration is often longer than in singular febrile convulsions. For this reason, we follow the guideline [Italian guideline: (CAPOVILLA *et al.*, 2009) ] - there is no current German AWMF guideline on the treatment of febrile convulsions. The only guideline that can be found is a non-updated guideline from the Society for Neuropaediatrics from 1999, which has since been removed from the AWMF server) recommends giving the child oxygen, keeping the airways open and waiting for the first 3 minutes, as the majority of seizures stop spontaneously within this time. If there is no end to the cerebral seizure after 3 minutes, diazepam should be administered at a dose of 0.5 mg/kg as a rectiole (liquid enema) (BERG *et al.*, 1996; KNUDSEN 2000). Diazepam administered rectally is rapidly absorbed and reaches anticonvulsant levels in the central nervous system after a further 3 minutes (CAPOVILLA *et al.*, 2009). In 44 children between 6 months and 5 years of age, rectal administration of diazepam was effective in interrupting febrile convulsions in 80% of cases (KNUDSEN 1979). This is a purely symptomatic therapy that is also used for epileptic seizures of various origins. The guidelines differ with regard to the length of the treatment-free interval before a benzodiazepine is administered. The Italian guidelines specify 3 minutes (CAPOVILLA *et al.*, 2009). The American Academy of Pediatrics (AAP) does not specify any fixed waiting times between the onset of the seizure and the start of drug therapy in its guidelines (AMERICAN ACADEMY OF PEDIATRICS 1996). In an addendum to this guideline, a waiting time of 5 minutes is assumed (WARDEN *et al.*, 2003) and reference is made to the treatment of status epilepticus (ALLDREDGE *et al.*, 2001; LOWENSTEIN *et al.*, 1998).

**Preclinical studies:** Schuchmann *et al.* (2006) developed a rat model in which young animals aged P8-P11 (corresponding approximately to the human age of 1-5 years) and P22-P23 (corresponding approximately to the human age of 12-18 years) were warmed up to a maximum temperature of 42°C in a heat chamber (SCHUCHMANN *et al.*, 2006). In the animals aged P8-11, cerebral seizures occurred in all animals (n=29). These were not seen in the older animals. The authors were able to show that the respiratory rate in the young animals increased disproportionately with the increase in body temperature (hyperventilation). This was accompanied by an increased exhalation of CO_2_, which caused respiratory alkalosis with a drop in intracerebral pH. It has long been known that such an alkalosis lowers the threshold for cerebral seizures in the brain and hyperventilation is therefore also used as a standard provocation procedure in EEG examinations. In order to restore the normal physiological state, Schuchmann *et al.* enriched the breathing air with 5% CO_2_, whereupon the pCO_2_ of the animals increased and the seizures stopped between 15 and 25 seconds. (SCHUCHMANN *et al.*, 2006)

In a further study, Tolner *et al.* (2011) were able to show that inhalation of 5% and 10% CO_2_ was also effective in suppressing cerebral seizures in a rat model (n=16) of myoclonic epilepsy (seizure induction by electrical stimulation) and in a non-human primate model (macaques, n=2, seizure induction by bicucillin instillation), and in significantly shortening the duration of seizures (TOLNER *et al.*, 2011).

## State of knowledge about the investigational medication

### Substance name, manufacturer, approval status, chemical and physical properties

**Substance name and approval status:** Carbogen is a gas mixture of 95% oxygen and 5% carbon dioxide, which is stored in pressurized gas cylinders. It is approved as a medicinal product and is mainly used in various diagnostic procedures such as transcranial Doppler sonography, SPECT examinations, for radiosensitization prior to radiotherapy and to improve cerebral blood flow. These areas of application are described in more detail below and supported by literature citations:

**Scintigraphic examination of the brain with TC-99m ECD (Tc-99m ethyl cysteinate):** Tc-99m ECD and SPECT can be used to examine regional cerebral blood flow *in vivo*, which can be reduced in various diseases such as stroke, arteriosclerosis of the cerebral arteries. Normally, an increase in pCO_2_ leads to an expansion of the cerebral vessels (perfusion reserve). To stimulate and measure this perfusion reserve, patients inhale carbogen. The resulting (relative) perfusion deficit in the areas of the brain supplied by the diseased vessels can thus be detected using Tc-99m ECD SPECT (ITO *et al.*, 2002; ITO *et al.*, 2003).

**Transcranial Doppler** sonography: The change in cerebral perfusion after carbogen inhalation can also be determined using transcranial Doppler sonography**.** This method is less complex and can be used in routine clinical diagnostics (ALVAREZ *et al.*, 2004).

**Improving the effectiveness of the response of tumors to radiation (radiosensitization) after inhalation of carbogen:** Siemann *et al.* (1975) were the first to show in an animal model that the radiosensitivity of tumors increased through the inhalation of carbogen and determined a minimum breathing time of 5-10 minutes before the start of radiation (SIEMANN *et al.*, 1975; SIEMANN *et al.*, 1977). This method is now standard for radiodiagnosis and therapy of certain types of tumors in humans (TAYLOR *et al.*, 2001).

**Improvement of neuroretinal function under systemic hyperoxia and hypercapnia:** Sponsel et al (1992) and Pakola (1993) were able to show that the inhalation of pure oxygen led to a vasoconstriction of the retinal vessels, while carbogen inhalation over 5 minutes led to an expansion of the retinal vessels and thus to an improvement in oxygen supply (PAKOLA *et al.*, 1993; SPONSEL *et al.*, 1992). In a similar study by Kergoat *et al.* (2004), all 18 individuals tolerated carbogen inhalation well and were able to calculate that the basal pH fell by 0.07 units during 5 minutes of inhalation (KERGOAT *et al.*, 2004).

**Improvement of cerebral perfusion in acute occlusion of the *carotid artery*:** Ashkanian *et al.* (2009) were able to show that inhalation of carbogen increased cerebral blood flow measured by PET in both healthy volunteers (n=10) and patients (n=6) with arterial occlusive disease of the *carotid artery*. However, the authors conclude that no general recommendation to treat patients with acute stroke with carbogen can be derived from these findings, as these findings would have to be randomized and verified in a larger cohort beforehand (ASHKANIAN *et al.*, 2008; ASHKANIAN *et al.*, 2009).

**Manufacturer:** MTI Industriegase AG in cooperation with Westphalen AG

**Medical test procedures:** The relevant documents can be found in the dossier for the test product.

### Side effects, tolerability, effects on physiological parameters

Physiologically, an increase in pCO_2_ in the blood leads to an increase in respiratory drive and respiratory rate of around 50-60% in healthy subjects (LEY 1991) and after prolonged inspiration (up to 25 minutes), some people may experience a subjective feeling of breathlessness (BAILEY *et al.*, 2005). This can lead to anxiety attacks and panic in a few predisposed individuals (COLASANTI *et al.*, 2008; GRIEZ *et al.*, 2007; LEY 1991). Griez *et al.* (2007) investigated the relationship between the triggering of such panic symptoms and the inhaled CO_2_ concentration (0, 9, 17.5 and 35%) in more detail in 64 healthy volunteers in a randomized double-blind study. There was no significant difference in the aggregated values of all cognitive symptoms between inhalation of room air and 9% CO2 in room air. Anxiety symptoms only occurred significantly more frequently at a CO_2_ concentration of 17.5% and above. There was no gender difference in the findings.

In addition to 5% CO_2_, the study medication also contains oxygen (O_2_) with a volume proportion of 95% in the *verum* and 100% in the *placebo*. In principle, oxygen can have a toxic effect through the formation of free oxygen radicals ("oxygen toxicity"). However, it should be noted that oxygen is only administered briefly over 3 minutes and not hyperbarically. Providing oxygen via a mask or nasal cannula (if available) would even be in line with the guidelines, as many children breathe irregularly during a febrile seizure and the oxygen saturation measured by pulse oximetry (SaO_2_) can fall below 90% (CAPOVILLA *et al.*, 2009). In everyday clinical practice, 100% humidified oxygen is used for this purpose at a flow rate of approx. 2-3 liters per minute. This would correspond exactly to the amount of oxygen administered from the cylinder. Clinical studies on oxygen toxicity in neurosurgical patients have also shown that breathing pure oxygen for 2 hours does not lead to oxygen toxicity if the markers *F_2_-isoprostane*, *protein sulfhydryl*, *glutathione level* (GSH) and *total radical scavenging capacity* (TRAP) are used to determine this (PUCCIO *et al.*, 2009) . The authors conclude from their measurement results: "[...] These preliminary findings suggest that brief periods of normobaric hyperoxia do not produce oxidative stress and/or change antioxidant reserves in CSF [...]".

### Pharmacokinetics

The gas components of the carbogen, oxygen (O_2_) and carbon dioxide (CO_2_), are absorbed from the air we breathe via the lungs. Both gases diffuse into the intravascular space via the alveolar and vascular endothelium. The diffusion coefficient of CO_2_ is lower than that of O_2_. On the other hand, the physical solubility of CO_2_ in serum is better than that of O_2_. This is due to the fact that CO_2_ is in equilibrium with sodium bicarbonate via the following reaction: CO_2_ + H_2_O + Na_2_CO_3_ →2 NaHCO_3_. The oxygen molecules are bound to hemoglobin during transport, while the CO_2_ is physically dissolved in the serum. The normal arterial CO_2_ partial pressure (paCO_2_) is physiologically between 30-40 mmHg.

The uptake and release of both gases depends on (1) the respiratory rate, (2) the tidal volume, (3) the pulmonary gas exchange surface, (4) the diffusion distance via the alveolar and capillary endothelium.

### Mechanism of action

The absorption of the CO_2_ contained in the carbogen leads to a slight increase in paCO_2_ and thus to a decrease in the intravascular and later also the intracerebral pH value. A decrease in the intracerebral pH value leads to a reduction in the excitability of central neurons and thus causes the antiepileptic effect (HELMY *et al.*, 2011; SCHUCHMANN *et al.*, 2009).

### Results of clinical trials conducted to date

**Initial results from individual treatment trials and systematic investigations during the pre-surgical evaluation of patients with epilepsy:** As early as 1928, Lennox *et al.* were able to show that the hypersynchronous activity in the EEG of patients with petit mal epilepsy could be suppressed by inhaling carbogen with a CO_2_ content of 10%. (LENNOX 1928; LENNOX *et al.*, 1936) The same was also seen in psychiatric patients in whom no seizures could be triggered after inhalation of 15-30% CO_2_ during electroconvulsive treatment. Similar findings of a centrally suppressive effect of inhaled CO_2_ were found in the cat, (POLLOCK 1949) in macaques (monkeys) (STEIN *et al.*, 1949) and confirmed in humans (GYARFAS *et al.*, 1949; POLLOCK *et al.*, 1949).

In the publication already mentioned above, Tolner *et al.* (2011) also investigated the influence of medical carbogen (5% CO_2_ + 95% O_2_) inhalation administered via breathing mask on the suppression of epileptiform discharges in the EEG or on the shortening of epileptic seizures in 7 patients between 14 and 52 years of age with severe focal epilepsy with psychomotor or secondary generalized seizures who were in a clinic for pre-surgical diagnosis. With carbogen inhalation, seizure duration was significantly (p<0. 027) shorter (76±9 s, n=7 seizures) than without inhalation (109±13 s, n=9 seizures). Due to the circumstances of the pre-surgical diagnostic monitoring, the carbogen application could not be used immediately after the onset of the seizure. The latency was between 23 and 74 s. Looking at the seizure duration after the start of carbogen inhalation, the following values were obtained: 32.1±6.6 s with and 65. 4±12.3 s without carbogen application (p=0.012 unpaired t-test, n=7).

As an alternative to carbogen inhalation, it would also be possible to use classic rebreathing (breathing into a plastic bag) to increase the pCO_2_ in the blood. This has also been used successfully in two patients with febrile convulsions under controlled conditions in an emergency department (published as an abstract in: Schuchmann *et al.* Suppression of complex febrile seizures by elevating respiratory CO_2_ using a rebreathing technique - two case reports. *Epilepsia* 50 (Suppl), 245, 2008). However, such rebreathing of exhaled air outside a clinic without the availability of the necessary monitoring equipment (pulse oximeter) by non-medically trained parents is impractical or potentially even dangerous, as it can lead to an excessive uncontrolled increase in CO_2_ partial pressure in the blood.

A much safer alternative is therefore the use of carbogen gas (95% O_2_ + 5% CO_2_). The high oxygen content ensures that asphyxia (suffocation) cannot occur and the carbon dioxide leads to the desired acidification of the blood, which then suppresses the epileptic discharges in the brain.

Based on the above-mentioned preliminary findings, we would now like to conduct a clinical study to test the efficacy of carbogen gas for interrupting acute febrile convulsions. If this therapy proves to be effective and safe, the administration of diazepam, which has many side effects, could be dispensed with in future, or diazepam could be administered after carbogen inhalation in an escalation scheme.

### Question and justification of the project (rationale)

**Carbogen is effective in stopping febrile convulsions in patients with febrile convulsions.**

Extensive data are available on the safety and tolerability of CARBOGEN in humans, on the basis of which it can be assumed that it is well tolerated. These data refer to adults. It is therefore necessary to investigate the safety and tolerability of this substance in children as well. The duration of application (3 minutes) in children is based on the results of the studies in adults.

# Aims of the clinical trial

The aim of this clinical trial is to test the effectiveness of a three-minute application of Carbogen (6 liters in total) using a low-pressure can with a breathing mask attached to interrupt febrile convulsions. Furthermore, it is to be tested whether the above-mentioned administration of Carbogen to children by their parents is safe and practicable in the home situation.

After 2 times 10 patients, all values of the patients included in the study up to that point are unblinded and analyzed by another statistician not involved in the other aspects of the study with regard to safety aspects and the interim analysis required for case number adjustment is performed. A medical expert decides whether the safety data allow the study to be continued. Using the method of Bauer Köhne (1994), the number of cases required for continuation is determined (see Section 10.5).

## Primary endpoint

**The primary endpoint of the study is:**

**[1]** **Efficacy** of carbogen inhalation determined by the number of patients with acute febrile convulsion in whom the test medication was used but was unsuccessful within the administration time interval of 3 minutes and standard therapy (rectal diazepam administration) was initiated. (Comparison of the *verum* group with the *placebo* group)

## Secondary endpoint

**[1]** **Safety** of carbogen inhalation measured by the number of adverse events

**[2]** Manageability of Carbogen low-pressure doses with attached breathing mask for interrupting febrile convulsions at home and on the move (mobility)

**[3]** Quality of life

**[4]** Satisfaction and anxiety of parents

## Study design

This is a monocentric, prospective, double-blind, *placebo*-controlled, randomized study. The study has three special features:

**[1]** Due to the limited evidence available at the present time, a two-phase adaptive design according to Bauer Köhne is used.

**[2]** Randomization cannot take place during the seizure, but rather upon inclusion in the study. However, as only 25% of all randomized children are expected to have a seizure, more patients must be randomized than can actually be included in the analysis. The evaluation population is therefore based on the principle of the "Modified ITT Population" (EMA, CPMP/EWP/558/95 rev2). This means that only children with seizures are analyzed for the ITT population.

A total of 288 children were recruited, 80 for the first and 208 for the second study phase. Seizures are expected in 25% of these children, so that a total of 20+52=72 children are included in the analysis. This number can be corrected upwards, but not downwards, after completion of the first study phase.

**[3]** After the first febrile convulsion, a crossover takes place, so that regardless of the result of the first febrile convulsion, patients with *verum* receive *placebo* for the next febrile convulsion and vice versa. For the subsequent febrile convulsions, all parents receive *verum* unblinded. Since it is foreseeable that a certain number of children will not suffer a second febrile convulsion during the study and therefore cannot be included in the crossover, this is not a true crossover study. "Crossover" and open label phase from the 3rd febrile convulsion are therefore evaluated as a secondary analysis.

## Schedule

The study will begin immediately after approval by the higher federal authority and a positive vote by the ethics committee. The recruitment period is 24 months, the treatment period 3 minutes. The study duration per participant is 24 months.

# Selection of patients

288 patients will be included. As the risk of recurrence after a febrile convulsion is approximately 25%, we will only include children who have already suffered at least one febrile convulsion.

## Inclusion criteria

The following inclusion criteria are mandatory:

**[1]** Condition after a febrile convulsion

**[2]** Age between 12 months and 5 years; after a positive result of the interim safety analysis according to Bauer & Köhne, infants from 6 months can also be included

**[3]** Written consent of the legal representatives of the minor (custodial parents)

## Exclusion criteria

The following exclusion criteria are mandatory:

**[1]** Serious other organ disease

**[2]** Detection of meningitis as a possible cause of the cerebral seizure

**[3]** Underlying neurological disease or brain malformation

**[4]** History of cerebral seizures without fever

**[5]** Detection of hypersynchronous activity in the EEG

**[6]** Disease of the respiratory tract (e.g. asthma)

**[7]** Further participation in a clinical study according to the AMG or MPG during the study

**[8]** Lack of consent of the person with custody for the storage and disclosure of pseudonymized disease data in the context of the clinical trial

**[9]** Known intolerance to carbogen

**[10]** Placement in an institution on the basis of an official or court order (e.g. in a children's home)

**[11]** Insufficient ability to consult with the legal guardians (e.g. due to lack of language skills)

# Treatment plan and study procedure

After the patient has been informed and has signed the informed consent form, the study begins for the patient. The legal guardians are given a can of the test product in accordance with the randomization list and are given practical training in the use of the can by the study doctor. Contents of the training are

1. General safety instructions (position the child on its side during the seizure, slightly extend the head backwards to prevent the tongue from falling back, interrupt the gas application immediately if the child vomits);
2. the activation and use of the can (only hold the mask loosely in front of the child as when inhaling and do not press it onto the face, remove the mask as soon as the rather loud flow noise is no longer audible after approx. 3 minutes), and
3. if the seizure continues after gas application, administer the standard medication (diazepam).

Education and training in the stable lateral position (to avoid airway obstruction by the tongue) is routine. Furthermore, the parents and the treating physicians are informed that the administration of antipyretic medication (e.g. paracetamol) or the application of physical measures to reduce fever (cool compresses) should be carried out **as in routine cases** and that the decision for or against fever reduction is **not** influenced by the study.

## Recruitment, screening and inclusion procedures

Patients are recruited via our outpatient clinics or after the first febrile seizure on the wards of our clinic. The parents or guardians are informed about the study by the attending physician, who is also the investigator of this study. After receiving verbal and written information, the legal guardians are given sufficient time to ask questions. If the legal guardians decide to participate, they and the investigator sign the informed consent form. If all inclusion criteria and no exclusion criteria are met, the study begins with the practical instruction of the legal guardians in the use of the carbogen pressure can by the investigator. Following this, the parents will be given a pressurized gas can with *verum* or *placebo*, depending on the study arm, in accordance with the randomization list. Randomization takes place via the label on the can or packaging. One *verum* and one *placebo* dose will be prepared per participant.

## Clinical examinations and ward round schedule

**[1]** Clinical neurological examination of the child including determination of neuromotor development, length, weight and head circumference at study inclusion and after each febrile seizure.

**[2]** The legal guardians are asked to contact the investigator by telephone as soon as a febrile convulsion has occurred at home and has been treated with the trial medication. The investigator will then make an appointment for a clinical visit at the NeuroCure Clinical Research Center. During this visit, the details of the febrile convulsion are recorded and possible adverse events are investigated. If it is not possible for the family to come to the Charité, home visits can also be made by the study physicians. Once the data collection has been completed, a second can of compressed gas is given to the participant's parents or guardians. In addition, parents will be made aware of the urgency of taking their child to a pediatrician or a pediatric emergency clinic after each febrile convulsion in order to rule out meningitis. If more than two seizures occur during the course of the study, parents will be given an unblinded dose of *verum* from the second seizure onwards.

**[3]** In addition to the clinical details, the caregivers are asked about practicability, quality of life, anxiety and mobility when administering the test product.

**[4]** Telephone visits every 6 months during the individual study period, if no seizures have occurred during this time interval.

### Description of the measures and deviation from the routine

**Clinical examination (internal and neurological):** During the clinical examination, weight, length, head circumference, blood pressure and heart rate are recorded and the lungs and abdomen are examined. The neurological examination includes the determination of vigilance, muscle strength, muscle tone, sensitivity, gait, cranial nerve status and coordination. At the end of the examination, it is recorded whether the child has developed according to age.

The specific data collected during the visit includes:

(1) Date and time of the seizure,

(2) Fever at the time of the seizure

(3) Maximum level of fever during the entire fever episode

(4) Cause of the febrile infection

(5) Did the febrile convulsion stop during inhalation of the test product?

(6) Did a diazepam rectiole have to be administered?

(7) Total duration of the seizure

(8) Focality of the seizure

The frequency of clinical visits depends on the number of seizure episodes. All these examinations correspond to routine medical practice.

## Description of the test medication

6 liters of carbogen gas (5% CO_2_ + 95% O_2_) are contained in the low-pressure can from MTI Industriegase AG (in cooperation with Westphalen AG). Together with the gas can, a disposable breathing mask with application lever and instructions for use are shrink-wrapped in the same plastic bag. The breathing mask is placed on the can and snaps into place. Now the lever on the breathing mask must be pressed and the gas begins to flow. The gas volume and valve size are designed so that the gas flows out completely in 3 minutes when the lever is pressed. The gas only flows when the lever is pressed.

The quantity of 6 liters of carbogen gas to be applied results from the physical flow conditions of the low-pressure gas cylinder and breathing mask unit. As carbogen is only administered over 3 minutes, the gas flow should build up a maximum of 5% CO2 in the mask over 3 minutes, which the child then breathes out. The actual amount of CO2 absorbed is then determined by the child's breathing rate and tidal volume. In reality, as shown in a ventilation model ( own unpublished data ), the pCO2 content increases to a maximum of 3% of the inhaled air (own unpublished data). However, this increase in inspiratory pCO2 is sufficient to raise the end-tidal pCO2 (which is regarded as a direct measure of pCO2 in the blood when lung function is intact) by up to 7 mmHg (own unpublished data).

The test products are manufactured, filled and labeled by the company MTI IndustrieGase AG. For further details, including blinding, see the detailed dossier on the test product.

### List of side effects and interactions

In all studies carried out to date on the inhalation of carbogen with 5% CO_2_, the gas has proven to be well tolerated and free or low in side effects. The side effects reported to date and associated with Carbogen were all mild.

The increase in pCO_2_ in the blood is physiologically associated with increased respiratory drive and can be accompanied by a slight feeling of suffocation (as when holding one's breath), especially when using tight-fitting breathing masks (BADDELEY *et al.*, 2000). In healthy test subjects, CO_2_ inhalation leads to an increase in respiratory rate of around 50-60% (LEY 1991) and after prolonged inhalation (up to 25 minutes), some people may experience a subjective feeling of breathlessness (BAILEY *et al.*, 2005). In 14 patients, Baddeley *et al.* (2000) determined the arterial CO_2_ partial pressure (paCO_2_) and found an average increase in paCO_2_ from 36.7±2.3 mmHg under air breathing to 45.1 ± 1.9 mmHg under breathing carbogen with 5% CO_2_. Even in premature infants, an increased paCO_2_ of 45-55 mmHg is used therapeutically as so-called "permissive hypercapnia" to protect the lungs and has been classified as safe and effective in various studies (MILLER *et al.*, 2007).

A double-blind, *placebo*-controlled study by Griez *et al.* (2007) examined the exact relationship between inhaled CO_2_ concentration (0, 9, 17.5 and 35% CO_2_) and the occurrence of 10 psychological symptoms in 64 healthy test subjects aged 35.8 ± 15.9 years (33 men, 31 women): (1) feeling cold, (2) nausea, (3) paresthesia, (4) sweating, (5) trembling, (6) palpitations, (7) chest pain, (8) shortness of breath, (9) feeling of suffocation, and (10) dizziness. From these 10 entities, they calculated an aggregated Sore, which did not differ significantly between inhalation of *placebo* and 9% CO_2_ and was only significantly increased from a CO_2_ concentration of 17.5% (GRIEZ *et al.*, 2007).

Since prolonged breathing of 5% CO_2_ triggers only a few physiological and psychological effects, Bailey *et al.* (2005) investigated long-term breathing of a higher concentration of 7.5% CO_2_ (in room air) over 20 minutes in n=21 healthy volunteers (6 women, 13 men) aged between 19 and 40 years in a *placebo*-controlled study. They found a significant increase in heart rate, blood pressure and the Panic Symptom Inventory (PSI) score, and concluded that this method, in contrast to breathing 5% CO_2,_ was suitable for modeling anxiety. Furthermore, the authors conclude that breathing 7.5% CO_2_ in healthy volunteers is safe and robustly and reproducibly triggers the effects described above.

With regard to the increase in paCO_2_ during carbogen inhalation in normal controls, the following should be noted: patients with febrile convulsions tend to hyperventilate, with a pathological drop in paCO_2_ to values that are too low (Schuchmann *et al.* *Epilepsia* 2011 - in press). In this patient population, carbogen inhalation would not increase paCO_2_ above the normal range, but would restore a physiological normal state. For this reason, the side effects mentioned above, which are occasionally observed in healthy volunteers, are even less likely to occur.

### Treatment scheme

**The test medication is dosed as follows:** Administration of 6 liters of carbogen (or oxygen in the *placebo* arm) from a low-pressure can via a breathing mask for 3 minutes. The dose content is calculated so that the 6 liters of gas flow out within the three minutes. Two blinded doses are prepared for each patient (one with the *verum* = carbogen, one with the *placebo* = oxygen). At the start of the study, the patient or their guardian is randomized to receive one of these two blinded doses to take home. If the patient suffers a seizure during their participation in the study and has used the dose, they will receive the corresponding other dose on their next visit to the center. After the second seizure, all patients will receive unblinded *verum* doses until the end of their participation in the study.

### Storage, issue and return

The delivery of the test medication by the company MTI (Neu-Ulm) is documented. The dispensing and return of the pressurized gas cans to the patients is also documented. Unused pressurized gas cans will be destroyed by the company at the end of the study.

## Concomitant medication / concomitant therapy / rescue medication

In general, necessary **concomitant medication/therapy** is permitted for the entire duration of the study (24 months for the individual participant). This is recorded in the CRF.

No standard medication is withheld from the patient, as far as one can assume a "standard therapy". There is no generally binding guideline in Germany (e.g. on the AWMF server). However, there is an Italian guideline (see investigator information) which recommends waiting 3 minutes for the seizures to stop spontaneously and then administering a diazepam rectiole if this does not occur. This is also the practice at the Charité

Therefore, the following procedure should be followed under study conditions:

1. As soon as the parents notice a febrile convulsion, the child should be given the study medication immediately. The gas flows out audibly for 3 minutes.
2. If the seizure does not stop after these 3 minutes, the parents should administer the diazepam rectiole (as usual in standard therapy). This administration then corresponds to a kind of "rescue medication".
3. If the seizure still does not stop, the parents should contact the emergency doctor.
4. In any case, parents should take a temperature after each seizure and present the child to the outpatient clinic.

The intake of the study medication is entered into the CRF by the study doctor at each visit.

Even though studies have shown that timely fever reduction has no influence on the recurrence of febrile convulsions, it should be expressly noted here that the use of fever-reducing measures does not have to be dispensed with as part of the study

## Blinding, emergency envelopes, unblinding

The blinding and emergency envelopes are carried out and prepared by the Charite pharmacy. The emergency envelopes are kept under lock and key at the trial center.

The study will be regularly unblinded for all patients after completion of all visits. Early unblinding is only permitted for safety reasons and must be documented and reported to the sponsor immediately.

# Risk-benefit assessment

**Preliminary remark:** this discussion of the risk-benefit balance contains only the most important clinical and pharmacological information relevant to the discussion of the topic. Carbogen is an approved medicinal product. Detailed information on the investigational product can be found in the protocol (see above), the dossier for the investigational product Carbogen, which is enclosed with this application.

The overall risk of the study is considered to be very low. The application of the carbogen quantity described above does not pose any risks because the administration of the carbogen only restores a physiological state and the laws of respiratory physiology rule out the possibility that the CO_2_ partial pressure in the arterial circulation (paCO_2_) could rise into the toxic range as a result of the administration of the test medication. The state of a study-related increased paCO_2_ lasts only a few minutes and returns to normal after 30 seconds after the end of the carbogen administration.

Irregular breathing can occur during a febrile seizure, as the diaphragm is also affected by the muscle twitching. The irregularity of breathing is therefore primarily a peripheral problem (tension of the diaphragm muscle during the seizure). However, the reduction in pCO_2_ during a febrile convulsion (as demonstrated by Schuchmann; Schuchmann *et al.* 2011) could also reduce the central respiratory drive. In this case, an increase in pCO_2_ caused by 5% CO_2_ administration would lead to an increase in the central respiratory drive and not to a slowing of breathing by restoring the natural physiological state. The cyanosis often seen is due to a lowered pO_2_ and this would be significantly increased by administering the 95% oxygen component of the carbogen. It is standard practice to give 100% oxygen to children who come to the emergency room while still having a seizure in order to ensure adequate oxygen saturation despite irregular breathing. Parents generally do not have the opportunity to administer such oxygen in the home environment. The 95% oxygen content of the carbogen would have the beneficial side effect for the child of reducing cyanosis and improving oxygen saturation. We therefore see no risk that the respiratory rate and cyanosis of the convulsing child could be exacerbated by the administration of carbogen - quite the opposite.

As no data on applicability, safety and tolerability in children are available to date, we will carry out an interim safety analysis according to Bauer & Köhne after 20 included events (seizures). A medical expert will then decide whether the safety data allow the study to be continued. Until a positive result is obtained, only children over the age of 12 months are included, i.e. no infants.

The parents are trained with the aid of a resuscitation manikin before the study medication is dispensed. The contents of the training are listed in Chapter 5. Furthermore, it was possible to test on an adult volunteer that rebreathing from the small-volume breathing mask only leads to a maximum 0.3% increase in the CO2 content of the inhaled air. This is due to the fact that the breathing mask intentionally does not seal tightly and no reservoir bag is used. In this respect, putting on the mask too tightly or for too long would not be dangerous. It is not technically possible to block the airways through incorrect use.

The scientists and doctors of the CARDIF study are of the opinion that a tightly sealed breathing mask (such as a resuscitation bag) should not be used in this study for the following reasons: **(i)** The child should only be provided with an atmosphere of 3-5% CO_2_ in front of its nasopharynx, from which it then actively inhales the corresponding quantities of gas. In this sense, the mask is designed more like an inhalation device and we have tested the gas flows at different breathing volumes and breathing frequencies with a corresponding resuscitation manikin of childhood. The desired CO_2_ concentrations could be maintained for 2-3 minutes at breathing rates and volumes of both an infant and a toddler after holding our standard mask in place (unpublished data). **(ii)** The use of a nose/mouth mask with a tight air seal (as in a resuscitation bag) would indeed be far too dangerous in the hands of medical laypersons, as it could lead to asphyxiation or aspiration if not used properly. In an experiment with an adult volunteer, the prolonged use of our preferred breathing mask, even without gas flow, did not lead to a drop in oxygen saturation measured by pulse oximetry for 350 seconds (as yet unpublished data). It is therefore intended that the mask should not be placed very close to the mouth and nose (but should only be used to create a certain gas atmosphere and *not generate excess pressure*).

No standard therapy is withheld from the patient. If the use of the study medication does not lead to the cessation of the seizure, the standard therapy (rectal administration of 5 mg diazepam) is used after 3 minutes in accordance with the guidelines. This means that the low-risk study intervention is only used in the time window in which no therapy would take place according to the guidelines.

Each individual seizure event is evaluated and documented by a member of the project team and the child is promptly seen and clinically examined in the study outpatient clinic. If the parents are unable to come to the study outpatient clinic, we offer the parents a home visit by a medical colleague. No study-related blood samples will be taken.

**Concluding statement:** Taking into account the planned area of application (febrile convulsions) and the preclinical and clinical data to date on carbogen in various areas of medical use in adults, it is to be expected that the potential benefit of this substance for the persons concerned (the possibility of rapid emergency therapy with few side effects and no sedation to interrupt febrile convulsions) far exceeds the potential risks, which are minor according to all the findings to date. Therefore, a clinical trial makes sense. We hereby assure that all information provided on the investigational product Carbogen has been researched with the greatest possible care and corresponds to the current state of knowledge at the time of application for this clinical trial. The risks of the study-related measures (physical examination without blood sampling and inhalation of Carbogen/oxygen via a mask) are to be considered minimal. We expect a positive effect of the *verum* on the earlier cessation of febrile convulsions.

# Termination and further treatment

## Premature discontinuation of a single patient

All legal guardians have the right to refuse further participation in the study at any time without giving reasons. The patient's participation must be discontinued immediately at the request of the legal guardian.

A patient may withdraw from the study at the discretion of the principal investigator. The reason for this should be fully documented in the study file. If the patient develops a condition during the course of the study that would have prevented their participation in the study according to the exclusion criteria, they must withdraw from the study immediately. The reasons for this should be fully documented in the study file. As soon as the continuation of the trial is no longer associated with the lowest possible risks and burdens (*verum* group) or minimal risks and burdens (*placebo* group) for the participant, the trial is discontinued for this participant.

In summary, the following criteria apply as termination criteria for the individual patient:

**[1]** Withdrawal of the consent of the custodian

**[2]** Subsequent occurrence of an exclusion criterion

**[3]** Occurrence of a SUSAR

**[4]** Unauthorized use of the study drug and non-compliance of the guardians (defined as at least two seizures without timely use of the study drug)

Note: if a patient misses more than two consecutive appointments without a reason that the principal investigator agrees with, then that patient is considered unavailable for follow-up.

## Premature termination of the entire clinical trial

The principal investigator may consider discontinuing the overall study if continuation is not justifiable. This is the case if more than 30% of the patients were discontinued prematurely for an individual reason. Furthermore, the entire trial is discontinued as soon as the continuation of the trial is no longer associated with the lowest possible risks and burdens (*verum* group) or minimal risks and burdens (*placebo* group) for the participants.

## Plan for further treatment after graduation/completion

The end of the study is the same as the end of treatment. Patients will continue to be treated by their regular pediatrician after the end of the study.

# Pharmacovigilance

## Definitions

**Adverse event (AE)**

An AE (adverse event) is defined as any adverse medical event in a patient in the context of a clinical trial, whereby the AE does not necessarily have to be related to the investigational medication or other measures during the clinical trial. An AE can therefore be any symptom or finding (e.g. laboratory value changes) that is temporally related to the intake of an investigational substance. All AEs are comprehensively documented in the Adverse Event CRF with symptomatology, duration (start/end), severity and suspected connection with the study medication. Diseases and symptoms that already existed before the start of the study medication are only classified as AEs if they worsen during treatment or require specific therapeutic intervention.

**Serious adverse event (SAE)**

An SAE is any adverse medical event, symptom or finding occurring during the study that

**[1]** Leads to death,

**[2]** is life-threatening

**[3]** requires inpatient hospital treatment or its extension,

**[4]** results in permanent and/or significant disability.

The study physician must take appropriate diagnostic and therapeutic measures to minimize the safety risk for the patient. The study physician should take appropriate measures to contribute to the clarification of a possible connection between the study medication and the SAE. Hospital treatments that were already planned before the start of the study are not formally classified, documented and reported as SAEs. Elective interventions or elective inpatient admissions for therapeutic or diagnostic purposes are also excluded from the SAE reporting requirement. All other SAEs will be reported immediately by the investigators to the sponsor.

**Suspected case of a suspected unexpected serious adverse reaction (SUSAR)**

A suspected unexpected serious adverse reaction (SUSAR) exists if serious adverse and unintended side effects are (temporally) related to the administration of the medicinal product and, after appropriate research, causes other than the administration of the medicinal product for the side effects can be excluded. SUSARs are reported to the higher federal authority (BfArM) and the Ethics Committee within the deadlines specified in the relevant regulations.

## Assessment of intensity

The investigator should classify AEs according to the following definitions:

**[1] Mild:** An AE is usually classified as mild if the patient finds the symptoms easily tolerable or less debilitating.

**[2] Moderate/moderate**: An AE is usually classified as moderately severe if the patient's activities and functions are limited but not completely impossible or eliminated.

**[3] Severe:** An AE is classified as severe if they are no longer able to carry out their usual activities.

## Assessment of the causal relationship

The following definitions are used to assess the relationship between the use of the test product and an AE:

**[1] None:** The time interval between the administration of the study medication and the occurrence of the AE excludes a causal relationship OR another cause for the AE has been found.

**[2] Unlikely:** The time interval between the administration of the study medication and the occurrence of the AE makes a causal relationship unlikely AND/OR the known effects of the study medication provide no evidence of causation of the AE and another cause is known that plausibly explains the AE AND/OR there may be some plausibility of a causal relationship based on the known effects of the study medication, but another cause is much more likely AND/OR another cause for the AE is confirmed and involvement of the study medication in the AE is unlikely.

**[3] Possible:** A certain plausibility of a causal relationship may exist due to the known effects of the study medication, but another cause must be considered at least as likely AND/OR although the known effects of the study medication do not suggest a relationship, there is no other adequate explanation for the AE.

**[4] Probable:** the pharmacologic properties of the study medication AND/OR the course of the AE AND/OR specific tests indicate involvement of the study medication, although other causes cannot be excluded with certainty.

**[5] Definitive:** The pharmacological properties of the study medication AND/OR the course of the AE AND/OR specific tests (laboratory tests etc.) indicate that the study medication is involved and other causes cannot be determined.

**[6] Not assessable:** An assessment of the correlation is not possible.

## Documentation of AEs and SAEs

Attention should be paid to AEs at all times during the study and targeted questions should be asked at every regular or unscheduled contact with participating patients. Any AEs must be documented in the CRF in as much detail as possible. This includes the following information:

**[1]** Symptoms, findings or diagnoses that characterize the AE; if possible, a diagnosis should be documented; if not (yet) possible, the patient's complaints and any abnormal examination findings (clinical, laboratory, etc.) should be documented.

**[2]** Start: Date and, if possible, also time

**[3]** Serious adverse event (SAE) (yes, no)

**[4]** Connection to the study medication

**[5]** Intensity (for definition see following subchapter)

**[6]** Pattern of occurrence (permanent, only after taking the study medication, etc.)

The following parameters are documented when the AE has ended, relevant changes occur, or at the end of the study at the latest:

**[1]** Any changes to the study medication by the investigator (e.g. no further doses are given after a seizure) and the reasons for this

**[2]** Drug treatment of AE

**[3]** Non-drug treatment of AE

**[4]** Connection to the study medication

**[5]** Status at the end of the AE (resolved, incompletely resolved, residual status, unchanged, unknown)

**[6]** Ongoing AE with relevant changes: Completion of a new AE-CRF

## Notification of SAEs by the auditor

The investigator informs the sponsor (here representative) immediately (within 24 hours) and in writing (SAE-CRF) about the occurrence of a serious adverse event. The report must be sent by fax to:

Dr. med. Ulrike Grieben
Charité - University Medicine Berlin
Experimental and Clinical Research Center
Lindenberger Weg 80, 13125 Berlin
Fax: 030 450 540 907

An SAE-CRF must be completed for each of these events and forwarded immediately to the address provided. If the required information is not available at this time, follow-up reports must be completed. For deaths, a copy of the autopsy report should be included if possible.

## Notification of (S)AEs and SUSARs by the sponsor

The sponsor (in this case the representative) will forward the documented (serious) adverse events to the competent higher federal authority and ethics committee on request.

The sponsor (here representative) reports every suspected case of an unexpected serious adverse reaction (SUSAR) of which he becomes aware to the responsible ethics committee and the responsible higher federal authority immediately, but at the latest within 15 days of becoming aware of it. Furthermore, he shall inform all investigators involved in the study.

In the event of a SUSAR that has led to a death or is life-threatening, the sponsor (here representative) shall immediately, but at the latest within 7 days of becoming aware of it, provide the responsible ethics committee and the responsible higher federal authority as well as all investigators involved with all information important for the assessment and within a maximum of 8 further days with the other relevant information.

The sponsor (here representative) shall inform the responsible ethics committee and the responsible higher federal authority immediately, but at the latest within 15 days of becoming aware of any facts that require a new review of the benefit-risk assessment of the investigational medicinal product. This includes in particular

**[1]** Individual case reports of expected serious side effects with an unexpected outcome

**[2]** Increase in the frequency of expected serious adverse events assessed as clinically relevant

**[3]** SUSARs that occurred after the subject had already completed the clinical trial (up to 3 months after the end/exclusion of the trial)

**[4]** Events related to the conduct of the study or the development of the investigational medicinal product that could potentially affect the safety of the subjects concerned

Personal data is always pseudonymized before it is transmitted. Before a SUSAR is reported, the blinding for this patient is removed.

## Expected adverse events

**Typical disease-associated AEs**: The disease is characterized by cerebral seizures in the context of febrile episodes. In rare cases, the seizures can lead to respiratory disorders, cerebral hypoxia and postictal paralysis (Todd's palsy), especially if they last longer than 15 minutes. In a population-based study, between 1.5-4.6% of children developed epilepsy after a first febrile convulsion. (ELLENBERG *et al.*, 1980)

**Medication-associated AEs:** (1) acute panic attack, (2) prologued hyperventilation

## Exception to the SAE definition

Excluded from the SAE definition and therefore not to be assessed, documented and reported as SAEs are inpatient treatments that were already planned before study inclusion and are therefore not related to the investigational medication.

## Documentation, storage of data and access to data

Patient documentation is made in ink or ballpoint pen; pencil entries are not made. Corrections are made as follows: The incorrect entry is crossed out with a simple line, the correct information is entered next to it and dated and signed off by the investigator.

The investigator keeps the original medical data at the study center. All information in the CRF must be traceable to the primary document (patient file). All essential study documents, e.g. correspondence with the ethics committee, monitoring authority, study management, study center, the signed consent forms, copies of the documentation forms (CRF) and the general study documentation (protocol, amendments) are kept at the study center for 10 years after completion of the clinical study. The original data of the study patients (medical records) must be stored in accordance with the archiving periods applicable at the study center.

The principal investigator and the study team have access to the original data and documents. Furthermore, the monitor will have access within the scope of his activities and the competent supervisory authority within the scope of an inspection. The participant will be informed about this (see patient information).

# Quality management

External GCP monitoring is carried out. The monitor regularly reviews the study documents during the course of the study and after the last study patient has completed treatment.

The monitor is responsible for verifying the conduct of the study in accordance with the study protocol together with the investigator. Confidentiality of the study documents, including patient data, must be ensured.

Some of the findings are documented directly in the study file and are therefore considered source data. The remaining findings are subjected to a source data check.

At least the following source data must be documented in the patient file:

**[1]** Demographic data of the patient (name, gender, date of birth)

**[2]** Date and signature under the declaration of consent

**[3]** Documentation of study inclusion and study/patient number

**[4]** Visit data of the patient

**[5]** Information on the inclusion and exclusion criteria

**[6]** Confirmation of the diagnosis

**[7]** SAEs and inpatient treatments (more detailed in the CRF)

**[8]** AEs (more detailed in the CRF)

**[9]** Application of study medication (start of treatment, interruption of treatment) and review of withdrawals and withdrawals

**[10]** Concomitant medication and concomitant diseases

**The respective CRF pages are the source for the following data:**

**[1]** Neurological and internal findings incl. functional scales

**[2]** Vital signs

**[3]** Date of the visits

# Statistical analysis

## Study design

This is a monocentric, prospective, double-blind, *placebo*-controlled, randomized study. The study has three special features:

**[1]** Due to the limited evidence available at the present time, a two-phase adaptive design according to Bauer Köhne is used.

**[2]** Randomization cannot take place during the seizure, but rather upon inclusion in the study. However, as only 25% of all randomized children are expected to have a seizure, more patients must be randomized than can actually be included in the analysis. The evaluation population is therefore based on the principle of the "Modified ITT Population" (EMA, CPMP/EWP/558/95 rev2). This means that only children with seizures are analyzed for the ITT population.

A total of 288 children were recruited, 80 for the first and 208 for the second study phase. Seizures are expected in 25% of these children, so that a total of 20+52=72 children are included in the analysis. This number can be adjusted upwards, but not downwards, after completion of the first study phase.

**[3]** After the first febrile convulsion, a crossover takes place, so that regardless of the result of the first febrile convulsion, patients with *verum* receive *placebo* for the next febrile convulsion and vice versa. For the subsequent febrile convulsions, all parents receive *verum* unblinded. Since it is foreseeable that a certain number of children will not suffer a second febrile convulsion during the study and therefore cannot be included in the crossover, this is not a true crossover study. "Crossover" and open label phase from the 3rd febrile convulsion are therefore evaluated as a secondary analysis.

## Target variables (endpoints)

**The primary endpoint of the study is:**

**[1]** The efficacy of carbogen inhalation determined by the number of patients with acute febrile convulsion in whom the test medication was used but was unsuccessful within the administration time interval of 3 minutes and standard therapy (rectal diazepam administration) was initiated. (Comparison of the *verum* group with the *placebo* group)

**Secondary endpoints are:**

**[1]** The safety of the application of 6 liters of carbogen via a low-pressure canister with a breathing mask to interrupt acute fiber convulsions in the home situation by parents.

**[2]** Manageability of Carbogen low-pressure doses with attached breathing mask for interrupting febrile convulsions at home and on the move (mobility)

**[3]** Quality of life

**[4]** Satisfaction and anxiety of parents

## Hypotheses

The aim is to prove the superiority of the experimental intervention (carbogen low pressure doses with a breathing mask to interrupt febrile convulsions in the home situation) vs. the control intervention (wait and see). The success rates under intervention and control are p_I_ and p_K_.

Null hypothesis: p_I_=p_K_

Alternative hypothesis: p_I_≠ _p(K)_

## Statistical evaluation methods

**Primary question:** The evaluation is carried out according to Bauer Köhne (1994) with〈 =0.025, c_〈_ =0.00380 and〈 _0_=0.5 (one-sided in each case). The main outcome diazepam administration no (success)/yes (failure) is evaluated as a binary endpoint using exact Fisher tests. The secondary endpoints "manageability" and "safety" are evaluated by relative frequency + two-sided 95% confidence interval. The other secondary endpoints are analyzed descriptively according to their scaling, p-values are given but are not to be interpreted as confirmatory. The dependence of repeated measurements ("crossover" and "open label" phase) is adjusted for a logistic regression model using generalized estimating equations.

## Determination of the optimum sample size (number of cases)

Overall, a significance level of 0.025 one-sided should be maintained. Assuming a difference of 75% vs. 25%, the power for the two-sided Fisher test is 86% for 2*26 patients (worst case p_1_=c /_〈_〈 _0_=0.00760). The actual power will probably be even higher, as the planned number of cases will only be increased but not decreased.

## Interim evaluations

An interim evaluation will occur after 2 times 10 patients. The results will possibly increase the number of cases, but not decrease.

## Definition of evaluation collectives

Primary evaluation population: Modified ITT population, only patients with seizures**.**

## Randomization, stratification and blinding

1:1 randomization in parallel group design, blinding achievable by providing *placebo* doses, no stratification

## Statistical data processing and biostatistical support of the study

Analyses are carried out with the help of SPSS for Windows, R and SAS by the Institute of Biometry and Clinical Epidemiology at Charité.

## Documentation

The documentation is carried out by the trial center.

# Ethical, legal and administrative aspects

## Legal aspects

The planning and execution of this study is subject to German legislation. The study may only begin once all legal provisions and requirements have been met. The study will be conducted in accordance with the ethical principles of the Declaration of Helsinki and the ICH-GCP guidelines as well as the AMG and data protection laws.

**Statement on the inclusion of minors:** According to the current state of medical science, we assume that the study medication is safe and tolerable and contributes to an earlier interruption of febrile convulsions compared to standard therapy. Febrile convulsions are a phenomenon of brain development in childhood. Therefore, by definition, they only occur in children between 6 months and 5 years of age, so that the above-mentioned questions cannot be examined in adult patients or older minors. The use of the study medication is medically indicated in these patients.

The study is associated with as few burdens and other foreseeable risks as possible. No standard therapy is withheld. Experience has shown that it is an explicit wish of the parents to use non-conventional medical approaches while avoiding sedating substances (e.g. diazepam or lorazepam). The regular review of the exposure limit, the child's well-being and a possible need for follow-up training of the parents is carried out by the study visits after a cerebral seizure or after each administration of the study drug and by telephone visits. More frequent study visits are intentionally not planned in order to avoid unnecessarily burdening the participants with travel. The discontinuation criteria are defined in such a way that any risks or burdens resulting from participation in the study are kept to a minimum. Should the investigator gain the impression from discussions with the parents and/or the child concerned or through observation that further participation in the study is not in the child's best interests, discontinuation of the study must be proactively addressed immediately.

The benefits of participating in the study outweigh the risks, as no study-related blood samples are taken and the patient is not deprived of standard therapy. The therapeutic offer to the child or its parents therefore remains completely unchanged by participating in the study.

Consent is given by the legal representative after he or she has been informed verbally and in writing. It must correspond to the presumed will of the minor, insofar as such a will can be ascertained. The patient or his/her representative will not be offered any financial or other material incentives to participate in a study.

The study is mandatory for the confirmation of the above hypotheses; studies on another patient group do not allow sufficient test results. The individual risk is low; we expect a benefit for the individual study participants. The group of children with febrile convulsions may benefit from the study results in that they may be offered an alternative treatment or an addition to the currently available therapies in the event of a positive outcome. The currently frequently used treatment with a benzodiazepine leads to prolonged sedation or loss of vigilance in a large proportion of children.

Please also see a separate statement in the appendix.

## Permits

The study protocol and any changes/amendments must be approved by the lead ethics committee of the State of Berlin and the Federal Institute for Drugs and Medical Devices BfArM in Bonn.

## Patient information and consent

The participating study physicians explain verbally and in writing the purpose and content of this study, the expected duration and the potential benefits and risks of participating in the study. The information is addressed to the legal representative of the minor (both custodial parents if available). The legal representative of the minor (both custodial parents, if any) will receive a version of the patient information and consent. The minor's legal representative (both custodial parents, if present) is given sufficient time and opportunity to ask questions. The legal representative of the minor (both custodial parents, if present) is informed that they can withdraw their consent at any time without giving reasons and without any disadvantages for the patient.

After an informative discussion, the legal representative of the minor (both custodial parents, if present) will be asked whether he/she wishes to participate in the study with his/her son/daughter and is willing to sign and personally date the patient consent form. Only if the legal representative of the minor (both custodial parents, if available) has voluntarily signed the patient consent form may the study be included. The patient consent form is then also signed and dated by the study physician*.*

The signed patient consent(s) remain(s) in the investigator's folder. The study physician documents on the corresponding CRF that the legal representative of the minor (both custodial parents if available) has signed the patient's consent. The legal representative of the minor (both custodial parents if available) receives a copy of the patient information and consent.

The patient information and consent will be amended as soon as new information in connection with the study or the study medication becomes known that could have an influence on the willingness of the minor's legal representative (both custodial parents if present) to participate. Likewise, the minor's legal representative (both custodial parents, if any) will be informed of any protocol amendments that result in a change to the patient information and consent. The legal representative of the minor (both custodial parents, if present) will be asked by the study physician whether he/she wishes to remain in the study with his/her son/daughter even after the changed situation and whether the custodial parent is willing to sign the amended patient consent form. Any change to the patient information and consent must first be approved by the ethics committee.

## Test person insurance

For possible damages caused by the study medication and by study-related measures, a volunteer insurance has been taken out with Chubb Insurance Company of Europe (Grafenberger Allee 295, 40237 Düsseldorf, Tel. 0211-87730, Fax 0211-8773333, insurance policy no. 99483310, sum insured per volunteer € 500,000.00). Each participant will receive a copy of the insurance conditions.

## Data protection

All patient-related data is recorded in pseudonymized form. Each patient is uniquely identified by a patient number or pseudonym, which is assigned upon inclusion. The investigator keeps a confidential patient list in which the patient numbers are linked to the full patient name. Only the local study team and the monitor have access to this list. The original files can be viewed by monitors, auditors and inspectors.

Legal guardians are informed that their child's disease-related data is stored in pseudonymized form and used for scientific evaluations (publications). The legal guardian has the right to be informed about the stored data of their child. The legal guardian is also informed that the pseudonymized data of their child may be passed on to the responsible higher federal authorities and the responsible ethics committees as part of the statutory reporting obligations on drug safety. Patients who do not consent to this disclosure may not participate in the study.

## Publication of the results of the study

It is intended to publish the study results in anonymous form. The study teams will be considered according to their participation.

# Bibliography

ALLDREDGE BK, Gelb AM, Isaacs SM, Corry MD, Allen F, Ulrich S, Gottwald MD, O'Neil N, Neuhaus JM, Segal MR, Lowenstein DH. A comparison of lorazepam, diazepam, and *placebo* for the treatment of out-of-hospital status epilepticus. *N Engl J Med* 2001;**345**:631-637.

ALVAREZ FJ, Segura T, Castellanos M, Leira R, Blanco M, Castillo J, Davalos A, Serena J. Cerebral hemodynamic reserve and early neurologic deterioration in acute ischemic stroke. *J Cereb Blood Flow Metab* 2004;**24**:1267-1271.

AMERICAN ACADEMY OF PEDIATRICS. Practice parameter: the neurodiagnostic evaluation of the child with a first simple febrile seizure. American Academy of Pediatrics. Provisional Committee on Quality Improvement, Subcommittee on Febrile Seizures. *Pediatrics* 1996;**97**:769-772.

ANNEGERS JF, Hauser WA, Shirts SB, Kurland LT. Factors prognostic of unprovoked seizures after febrile convulsions. *N Engl J Med* 1987;**316**:493-498.

ASHKANIAN M, Borghammer P, Gjedde A, Ostergaard L, Vafaee M. Improvement of brain tissue oxygenation by inhalation of carbogen. *Neuroscience* 2008;**156**:932-938.

ASHKANIAN M, Gjedde A, Mouridsen K, Vafaee M, Hansen KV, Ostergaard L, Andersen G. Carbogen inhalation increases oxygen transport to hypoperfused brain tissue in patients with occlusive carotid artery disease: increased oxygen transport to hypoperfused brain. *Brain Res* 2009;**1304**:90-95.

BADDELEY H, Brodrick PM, Taylor NJ, Abdelatti MO, Jordan LC, Vasudevan AS, Phillips H, Saunders MI, Hoskin PJ. Gas exchange parameters in radiotherapy patients during breathing of 2%, 3.5% and 5% carbogen gas mixtures. *Br J Radiol* 2000;**73**:1100-1104.

BAILEY JE, Argyropoulos SV, Kendrick AH, Nutt DJ. Behavioral and cardiovascular effects of 7.5% CO2 in human volunteers. *Depress Anxiety* 2005;**21**:18-25.

BAUER P, Kohne K. Evaluation of experiments with adaptive interim analyses. *Biometrics* 1994;**50**:1029-1041.

BAUMANN RJ, Duffner PK. Treatment of children with simple febrile seizures: the AAP practice parameter. American Academy of Pediatrics. *Pediatr Neurol* 2000;**23**:11-17.

BERG AT. Febrile seizures and epilepsy: the contributions of epidemiology. *Paediatr Perinat Epidemiol* 1992;**6**:145-152.

BERG AT, Shinnar S. Complex febrile seizures. *Epilepsia* 1996;**37**:126-133.

BERG AT, Shinnar S, Darefsky AS, Holford TR, Shapiro ED, Salomon ME, Crain EF, Hauser AW. Predictors of recurrent febrile seizures. A prospective cohort study. *Arch Pediatr Adolesc Med* 1997;**151**:371-378.

CAPOVILLA G, Mastrangelo M, Romeo A, Vigevano F. Recommendations for the management of "febrile seizures": Ad Hoc Task Force of LICE Guidelines Commission. *Epilepsia* 2009; **50 Suppl 1**:2-6.

COLASANTI A, Salamon E, Schruers K, van DR, van DM, Griez EJ. Carbon dioxide-induced emotion and respiratory symptoms in healthy volunteers. *Neuropsychopharmacology* 2008;**33**:3103-3110.

ELLENBERG JH, Nelson KB. Sample selection and the natural history of disease. Studies of febrile seizures. *JAMA* 1980;**243**:1337-1340.

GRIEZ EJ, Colasanti A, van DR, Salamon E, Schruers K. Carbon dioxide inhalation induces dose-dependent and age-related negative affectivity. *PLoS One* 2007;**2** :e987.

GYARFAS K, POLLOCK GH, STEIN SN. Central inhibitory effects of carbon dioxide; convulsive phenomena. *Proc Soc Exp Biol Med* 1949;**70**:292.

HELMY MM, Tolner EA, Vanhatalo S, Voipio J, Kaila K. Brain alkalosis causes birth asphyxia seizures, suggesting therapeutic strategy
1. *Ann Neurol* 2011.

ITO H, Kanno I, Ibaraki M, Hatazawa J. Effect of aging on cerebral vascular response to Paco2 changes in humans as measured by positron emission tomography. *J Cereb Blood Flow Metab* 2002;**22**:997-1003.

ITO H, Kanno I, Ibaraki M, Hatazawa J, Miura S. Changes in human cerebral blood flow and cerebral blood volume during hypercapnia and hypocapnia measured by positron emission tomography. *J Cereb Blood Flow Metab* 2003;**23**:665-670.

KERGOAT H, Tinjust D. Neuroretinal function during systemic hyperoxia and hypercapnia in humans. *Optom Vis Sci* 2004;**81**:214-220.

KNUDSEN FU. Rectal administration of diazepam in solution in the acute treatment of convulsions in infants and children. *Arch Dis Child* 1979;**54**:855-857.

KNUDSEN FU. Febrile seizures: treatment and prognosis. *Epilepsia* 2000;**41**:2-9.

LENNOX WG. The effect of on epilptic seizures of varying the composition of the respired air. *J Clin Invest* 1928;**6**:23-24.

LENNOX WG, Gibbs FA, Gibbs EL. Effect on the electro-encephalogram of drugs and conditions which influence seizures. *Arch Neurol Psychiatry* 1936;**36**:1236-1245.

LEY R. Ventilatory control of heart rate during inhalation of 5% CO2 and types of panic attacks. *J Behav Ther Exp Psychiatry* 1991;**22**:193-201.

LOWENSTEIN DH, Alldredge BK. Status epilepticus. *N Engl J Med* 1998;**338**:970-976.

MILLER JD, Carlo WA. Safety and effectiveness of permissive hypercapnia in the preterm infant. *Curr Opin Pediatr* 2007;**19**:142-144.

NELSON KB, Ellenberg JH. Predictors of epilepsy in children who have experienced febrile seizures. *N Engl J Med* 1976;**295**:1029-1033.

OFFRINGA M, Bossuyt PM, Lubsen J, Ellenberg JH, Nelson KB, Knudsen FU, Annegers JF, el-Radhi AS, Habbema JD, rksen-Lubsen G, . Risk factors for seizure recurrence in children with febrile seizures: a pooled analysis of individual patient data from five studies. *J Pediatr* 1994;**124**:574-584.

PAKOLA SJ, Grunwald JE. Effects of oxygen and carbon dioxide on human retinal circulation. *Invest Ophthalmol Vis Sci* 1993;**34**:2866-2870.

POLLOCK GH. Central inhibitory effects of carbon dioxide; Felis domesticus. *J Neurophysiol* 1949;**12**:315-324.

POLLOCK GH, STEIN SN, GYARFAS K. Central inhibitory effects of carbon dioxide; man. *Proc Soc Exp Biol Med* 1949;**70**:291.

PUCCIO AM, Hoffman LA, Bayir H, Zullo TG, Fischer M, Darby J, Alexander S, Dixon CE, Okonkwo DO, Kochanek PM. Effect of short periods of normobaric hyperoxia on local brain tissue oxygenation and cerebrospinal fluid oxidative stress markers in severe traumatic brain injury. *J Neurotrauma* 2009;**26**:1241-1249.

SCHNAIDERMAN D, Lahat E, Sheefer T, Aladjem M. Antipyretic effectiveness of acetaminophen in febrile seizures: ongoing prophylaxis versus sporadic usage. *Eur J Pediatr* 1993;**152**:747-749.

SCHUCHMANN S, Schmitz D, Rivera C, Vanhatalo S, Salmen B, Mackie K, Sipila ST, Voipio J, Kaila K. Experimental febrile seizures are precipitated by a hyperthermia-induced respiratory alkalosis. *Nat Med* 2006;**12**:817-823.

SCHUCHMANN S, Vanhatalo S, Kaila K. Neurobiological and physiological mechanisms of fever-related epileptiform syndromes. *Brain Dev* 2009;**31**:378-382.

SIEMANN DW, Bronskill MJ, Hill RP, Bush RS. The relationship between mouse arterial partial pressure of oxygen (PaO2) and the effectiveness of localized tumour irradiation. *Br J Radiol* 1975;**48**:662-667.

SIEMANN DW, Hill RP, Bush RS. The importance of the pre-irradiation breathing times of oxygen and carbogen (5% CO2: 95% O2) on the in vivo radiation response of a murine sarcoma. *Int J Radiat Oncol Biol Phys* 1977;**2**:903-911.

SPONSEL WE, DePaul KL, Zetlan SR. Retinal hemodynamic effects of carbon dioxide, hyperoxia, and mild hypoxia. *Invest Ophthalmol Vis Sci* 1992;**33**:1864-1869.

STEIN SN, POLLOCK GH. Central inhibitory effects of carbon dioxide; Macacus rhesus. *Proc Soc Exp Biol Med* 1949;**70**:290.

TAYLOR NJ, Baddeley H, Goodchild KA, Powell ME, Thoumine M, Culver LA, Stirling JJ, Saunders MI, Hoskin PJ, Phillips H, Padhani AR, Griffiths JR. BOLD MRI of human tumor oxygenation during carbogen breathing. *J Magn Reson Imaging* 2001;**14**:156-163.

TOLNER EA, Hochman DW, Hassinen P, Otahal J, Gaily E, Haglund MM, Kubova H, Schuchmann S, Vanhatalo S, Kaila K. Five percent CO is a potent, fast-acting inhalation anticonvulsant. *Epilepsia* 2011;**52**:104-114.

WARDEN CR, Zibulewsky J, Mace S, Gold C, Gausche-Hill M. Evaluation and management of febrile seizures in the out-of-hospital and emergency department settings. *Ann Emerg Med* 2003;**41**:215-222.

# Attachments

**Appendix**  Statement on gender distribution

**Appendix**  Statement on further treatment after the end of the study

**Appendix C** Benefit-risk assessment incl. statement on the inclusion of minors

**Appendix A**

**Statement on gender distribution (according to GCP-V § 7 (2) No. 12)**

Patients of both sexes are included, as no sex-specific differences in the efficacy and safety of the drug to be tested are to be expected (see GCP-V § 7 (2) No. 12) and the incidence of febrile convulsions is the same in both sexes.

**Appendix B**

**Statement on further treatment after the end of the study (according to GCP-V § 7 (2) No. 13)**

After 24 months, the double-blind, *placebo*-controlled study ends for the patient. After the end of the study, patients continue to be treated by their regular pediatrician or outpatient clinic.

**Appendix C**

**Risk-benefit assessment incl. statement on the inclusion of minors**

**Preliminary remark:** This discussion of the risk-benefit balance contains only the most important clinical and pharmacological information relevant to the discussion of the topic. Carbogen is an approved medicinal product. Detailed information on the investigational product can be found in the protocol (see above), the dossier for the investigational product Carbogen, which is enclosed with this application.

The overall risk of the study is considered to be very low. The application of the carbogen quantity described above does not pose any risks because the administration of the carbogen only restores a physiological state and the laws of respiratory physiology rule out the possibility that the CO_2_ partial pressure in the arterial circulation (paCO_2_) could rise into the toxic range as a result of the administration of the test medication. The state of a study-related increased paCO_2_ lasts only a few minutes and returns to normal after 30 seconds after the end of the carbogen administration.

In addition to 5% CO_2_, the study medication also contains oxygen (O_2_) with a volume proportion of 95% in the *verum* and 100% in the *placebo*. In principle, oxygen can have a toxic effect through the formation of free oxygen radicals ("oxygen toxicity"). However, it should be noted that oxygen is only administered briefly over 3 minutes and not hyperbarically. Providing oxygen via a mask or nasal cannula (if available) would even be in line with the guidelines, as many children breathe irregularly during a febrile seizure and the oxygen saturation measured by pulse oximetry (SaO_2_) can fall below 90% (CAPOVILLA *et al.*, 2009) . In everyday clinical practice, 100% humidified oxygen is used for this purpose at a flow rate of approx. 2-3 liters per minute. This would correspond exactly to the amount of oxygen administered from the bottle. Parents would therefore also have the option of administering oxygen in the home environment and could counteract cyanosis. Clinical studies on oxygen toxicity in neurosurgical patients have also shown that breathing pure oxygen for 2 hours does not lead to oxygen toxicity if the markers *F_2_-isoprostane*, *protein sulfhydryls*, *glutathione levels* (GSH) and *total radical scavenging capacity* (TRAP) are used to determine this (PUCCIO *et al.*, 2009). The authors conclude from their measurement results: "[...] These preliminary findings suggest that brief periods of normobaric hyperoxia do not produce oxidative stress and/or change antioxidant reserves in CSF [...]".

Peripheral cyanosis can occur as part of a febrile convulsion. This is mainly caused by irregular breathing, as the diaphragm is also affected by the muscle twitching. The irregularity of breathing is therefore primarily a peripheral problem (tension of the diaphragm muscle during the seizure). However, the reduction in pCO_2_ during a febrile convulsion (as demonstrated by Schuchmann; Schuchmann *et al.* 2011) could also reduce the central respiratory drive. In this case, an increase in pCO_2_ caused by 5% CO_2_ administration would lead to an increase in the central respiratory drive and not to a slowing of breathing by restoring the natural physiological state. The cyanosis often seen is due to a lowered pO_2_ and this would be significantly increased by administering the 95% oxygen component of the carbogen. It is standard practice to give 100% oxygen to children who come to the emergency room while still having a seizure in order to ensure adequate oxygen saturation despite irregular breathing. Parents generally do not have the opportunity to administer such oxygen in the home environment. The 95% oxygen content of the carbogen would have the beneficial side effect for the child of reducing cyanosis and improving oxygen saturation. We therefore see no risk that the respiratory rate and cyanosis of the convulsing child could be exacerbated by the administration of carbogen - quite the opposite.

As no data on applicability, safety and tolerability in children are available to date, we will carry out an interim safety analysis according to Bauer & Köhne after 20 included events (seizures). A medical expert will then decide whether the safety data allow the study to be continued. Until a positive result is obtained, only children over the age of 12 months are included, i.e. no infants.

The parents are trained with the aid of a resuscitation manikin before the study medication is dispensed. The contents of the training are listed in Chapter 5. Furthermore, it was possible to test on an adult volunteer that rebreathing from the small-volume breathing mask only leads to a maximum 0.3% increase in the CO_2_ content of the inhaled air. This is due to the fact that the breathing mask does not intentionally seal tightly and no reservoir bag is used. In this respect, putting on the mask too tightly or for too long would not be dangerous. It is not technically possible to block the airways through incorrect use.

The scientists and doctors of the CARDIF study are of the opinion that a tightly sealed breathing mask (such as a resuscitation bag) should not be used in this study for the following reasons: **(i)** The child should only be provided with an atmosphere of 3-5% CO_2_ in front of its nasopharynx, from which it then actively inhales the corresponding quantities of gas. In this sense, the mask is designed more like an inhalation device and we have tested the gas flows at different breathing volumes and breathing frequencies with a corresponding resuscitation manikin of childhood. The desired CO_2_ concentrations could be maintained for 2-3 minutes at breathing rates and volumes of both an infant and a toddler after holding our standard mask in place (unpublished data). **(ii)** The use of a nose/mouth mask with a tight air seal (as in a resuscitation bag) would indeed be far too dangerous in the hands of medical laypersons, as it could lead to asphyxiation or aspiration if not used properly. In an experiment with an adult volunteer, the prolonged holding of the breathing mask favored by us, even without gas flow, did not lead to a drop in the oxygen saturation measured by pulse oximetry for 350 seconds (own unpublished data). It is therefore intended that the mask should not be placed very close to the mouth and nose (but should only be used to create a certain gas atmosphere and *not generate excess pressure*).

No standard medication is withheld from the patient, as far as one can assume a "standard therapy". There is no generally binding guideline in Germany (e.g. on the AWMF server). However, there is an Italian guideline (see investigator information) which recommends waiting 3 minutes for the seizures to stop spontaneously and then administering a diazepam rectiole if this does not occur. This is also the practice at the Charité. If the use of the study medication does not lead to the cessation of the seizure, the "standard therapy" (rectal administration of 5 mg diazepam) is used after 3 minutes in accordance with the guidelines. This means that the low-risk study intervention is only used in the time window in which no therapy would take place according to the guidelines.

Each individual seizure event is evaluated and documented by a member of the project team and the child is promptly seen and clinically examined in the study outpatient clinic. If the parents are unable to come to the study outpatient clinic, we offer the parents a home visit by a medical colleague. No study-related blood samples will be taken.

**Concluding statement:** Taking into account the planned area of application (febrile convulsions) and the preclinical and clinical data to date on carbogen in various areas of medical use in adults, it is to be expected that the potential benefit of this substance for the persons concerned (the possibility of rapid emergency therapy with few side effects and no sedation to interrupt febrile convulsions) far exceeds the potential risks, which are minor according to all the findings to date. Therefore, a clinical trial makes sense. We hereby assure that all information provided on the investigational product Carbogen has been researched with the greatest possible care and corresponds to the current state of knowledge at the time of application for this clinical trial. The risks of the study-related measures (physical examination without blood sampling and inhalation of Carbogen/oxygen via a mask) are to be considered minimal. We expect a positive effect of the *verum* on the earlier cessation of febrile convulsions

**Statement on the inclusion of minors:** the special regulations for studies involving minors set out below in Section 40 of the German Medicines Act are complied with in the CARDIF study:

*§Section 40 (4) No. 1 AMG: The medicinal product must be intended for the detection or prevention of diseases in minors and the use of the medicinal product must be indicated according to the findings of medical science in order to detect diseases in minors or to protect them from diseases. The medicinal product is indicated if its use in the minor is medically indicated.*

The use of carbogen administration in minors is indicated to protect them from prolonged cerebral seizures (prevention of illness). Standard therapy (diazepam) is not withheld from children, as it should only be administered after 5 minutes according to the guidelines. As carbogen inhalation restores a normal physiological state, we assume that less adverse drug effects (sedation) occur with carbogen inhalation than with diazepam administration.

*§Section 40 (4) No. 2 AMG: Clinical trials on adults or other research methods must not be expected to produce adequate test results according to the findings of medical science.*

Febrile seizures do not occur in adults, as they are a developmental phenomenon of the child's brain and by definition only occur between the ages of 6 months and 6 years. Acute carbogen administration to interrupt seizures can therefore not be tested in an adult population.

*§Section 40 (4) no. 3 AMG: Consent is given by the legal representative after he or she has been informed in accordance with paragraph 2. It must correspond to the presumed will of the minor, insofar as such a will can be ascertained. Before the start of the clinical trial, the minor shall be informed by an investigator experienced in dealing with minors about the trial, the risks and the benefits, insofar as this is possible in view of his or her age and mental maturity; if the minor declares that he or she does not wish to participate in the clinical trial or expresses this in any other way, this shall be taken into account. If the minor is capable of recognizing the nature, significance and scope of the clinical trial and is able to express his/her will accordingly, his/her consent is also required. In addition to the legal representative, the minor must also be given the opportunity for a consultation in accordance with subsection 2 sentence 2.*

The consent of the legal representatives is a prerequisite for participation in the study. The child's will is taken into account as soon as the child is at an age at which it can be determined. The study also involves the inhalation of a gas mixture in unconscious children during a cerebral seizure. It can be assumed that the child is considerably more stressed by the ongoing seizure than by the 3-minute carbogen inhalation from a breathing mask held loosely in front of the child's face. The investigators are requested in the protocol to ask the parents for and document the exact details of the seizure in close temporal proximity (on the same or the following day) and thus also to record the child's exposure.

*§Section 40 (4) No. 4 AMG: The clinical trial may only be conducted if it is associated with as little stress and other foreseeable risks as possible for the person concerned; both the degree of stress and the risk threshold must be specifically defined in the trial protocol and constantly reviewed by the investigator.*

The study is associated with as few stresses and other foreseeable risks as possible. No standard therapy is withheld.

We consider the strain on Carbogen's breathing caused by a breathing mask to be low, as it is not associated with pain, undesirable side effects or similar inconveniences for the child. In this respect, we would consider the rectal administration of diazepam as a microclyster to be more stressful for a child. We have now introduced a passage in the protocol that explicitly asks the investigators to check these points (degree of stress and risk) after each administration of the investigational product.

In addition, parents receive intensive training on how to use the compressed gas cylinder and breathing mask. Parents are only ever given a single can to take home. In the worst case, both unintentional and intentional misuse of the study medication can only lead to the child not inhaling enough carbogen. An overdose or misuse is not possible. Use is also harmless in the event of a seizure mistakenly identified as a febrile convulsion. The application in the event of a febrile convulsion lasts 3 minutes. It can be discontinued at any time. The effect of the medication ends immediately after discontinuation. There are no purely study-related risks, as the application of the dose and the breathing of Carbogen do not involve any risks. The only study-related burden is that the parents have to take care of the application of the can in a stressful situation. Attempts are made to alleviate this additional burden through intensive personal training and information material for parents. However, experience has shown that all parents would like to have a meaningful activity during the seizure. Many parents state that the helpless and inactive watching and waiting until diazepam can be administered is very stressful.

The regular review of the exposure limit, the child's well-being and a possible need for retraining of the parents is carried out by the study visits after a cerebral seizure or after each administration of the investigational product and by telephone visits. More frequent study visits are intentionally not planned in order to avoid unnecessarily burdening the participants with travel. The discontinuation criteria are defined in such a way that any risks or burdens resulting from participation in the study are kept to a minimum. Should the investigator gain the impression from discussions with the parents and/or the child concerned or through observation that further participation in the study is not in the child's best interests, discontinuation of the study must be proactively addressed immediately. The benefits of study participation outweigh the risks, as no study-related blood samples are taken and the patient is not deprived of standard therapy. The therapeutic offer to the child or its parents (e.g. fever reduction and possibly diazepam administration) therefore remains completely unchanged by participation in the study.

Furthermore, an interim safety analysis according to Bauer & Köhne is carried out after 10 patients.

*§Section 40 (4) no. 5 AMG: Benefits with the exception of reasonable compensation may not be granted*

No benefits are granted. Only the travel costs to the study center can be reimbursed at the parents' request.
