# Supplementary material for: The use of carbogen for interruption of febrile seizures - the randomized controlled CARDIF trial
Source: PLoS One. 2025 Dec 23;20(12):e0324422. doi: 10.1371/journal.pone.0324422 (PMC12725555; doi:10.1371/journal.pone.0324422)
Supplement: S2 File — (PDF) [file pone.0324422.s002.pdf]

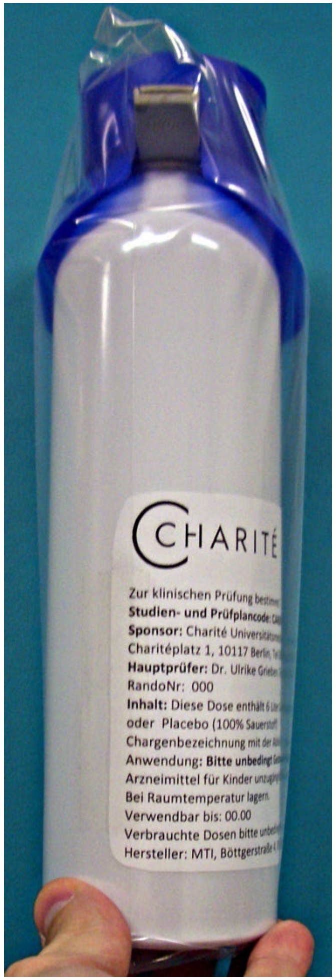

Study medication in its original packaging with blue breathing mask

1. Tear open and remove the plastic packaging foil.

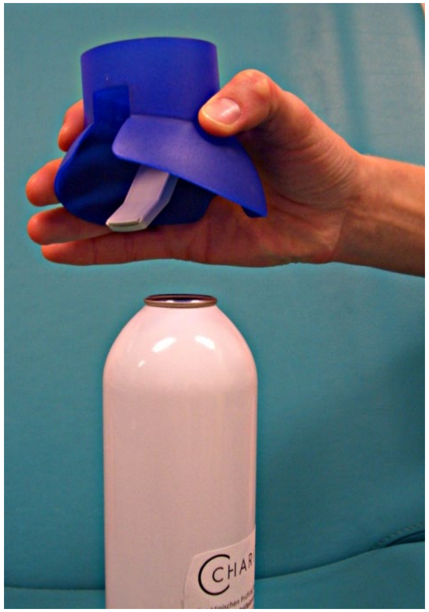

2. Lift off the mask

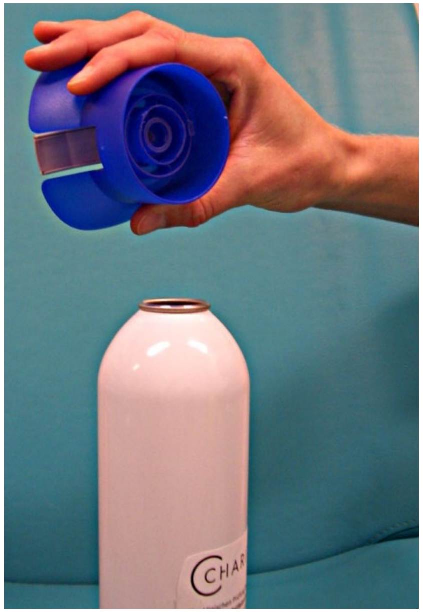

3. Turn the mask around

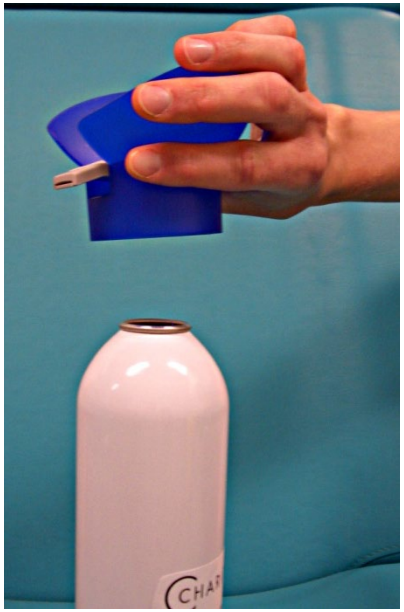

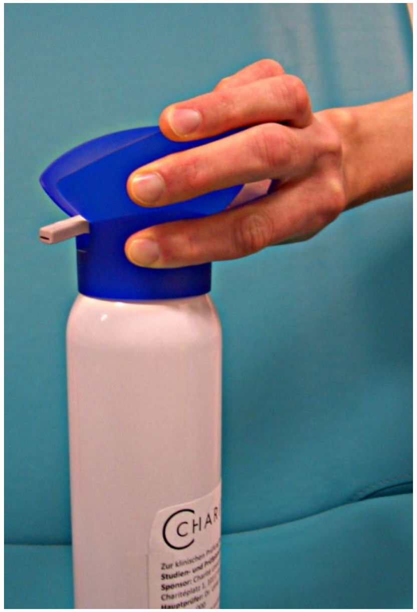

4. Place the mask firmly on the bottle **until it clicks into place**

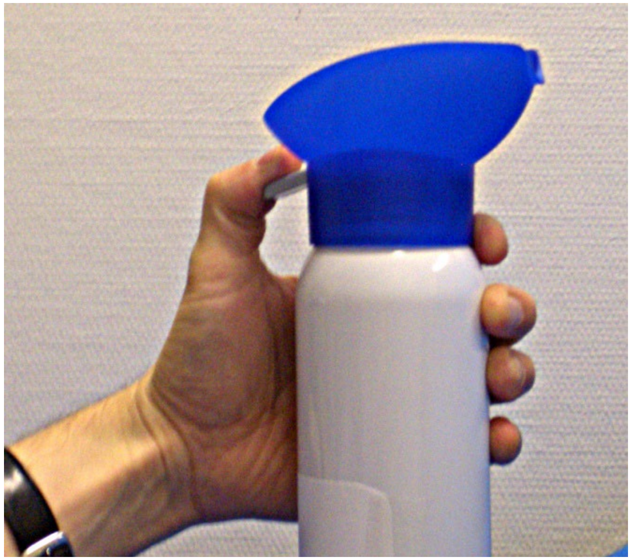

5. Hold the bottle as shown. When pressure is applied to the gray lever, gas will begin to flow into the mask.

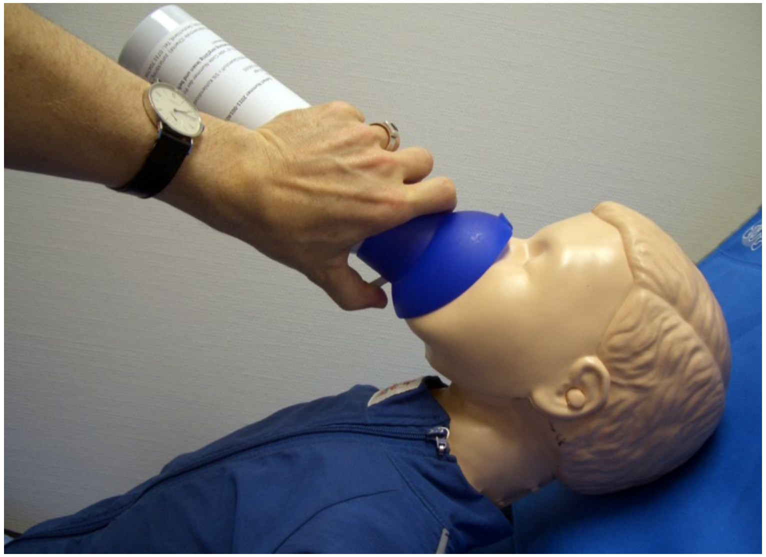

6. Hold the mask in front of the the child's face. **Do not press the mask on the face! The gray lever on the mask must be pressed down throughout the entire time (3 minutes).**
