# Supplementary material for: The use of carbogen for interruption of febrile seizures - the randomized controlled CARDIF trial
Source: PLoS One. 2025 Dec 23;20(12):e0324422. doi: 10.1371/journal.pone.0324422 (PMC12725555; doi:10.1371/journal.pone.0324422)
Supplement: Attachment — Submitted filename: 20250918_response_to_reviewers.docx [file pone.0324422.s006.docx]

**Reviewer #1**

The authors have addressed all my previous comments and the content of the revised manuscript has improved significantly

**Response:** We thank the reviewer for the effort to read and suggest improvements to our manuscript.

**Reviewer #2**

[1] What proportion of the patients in your study had complex febrile seizures, and what percentage had simple febrile seizures?

**Response:** Of the total number of seizures, n = 32 were simple febrile seizures and n = 6 were complex febrile seizures. Of those treated with verum, n = 12 were simple and n = 3 were complex febrile seizures; all n = 11 febrile seizures treated with placebo were simple febrile seizures. The n = 3 complex febrile seizures in the verum arm were not interrupted by verum. We have now entered this information into the CONSORT flow chart.

The difference in complex *versus* simple febrile seizures could indeed partly explain the negative outcome of the study. We thank the reviewer for this important comment. We therefore added this finding to the result section (“In the verum arm n = 12 recurrent febrile seizures were simple and n = 3 were complex, whereas all recurrent febrile seizures in the placebo arm were simple.”) and a sentence to the discussion section (“Furthermore, the difference in complex *versus* simple febrile seizures in both study arms may have influenced the outcome as complex febrile seizures only occurred in the verum arm.”).

[2] Using parent-reported seizure duration, benzodiazepine use, and any serious adverse events is understandable, but wouldn't it be better to also add home video recording since at times patients can have a seizure attack but the parents may not notice it if they are not around, or if they are sleeping.

**Response:** Indeed it would have been ideal to have some video footage. However, first we have to consider that the study was conducted ten years ago when mobile phones with video capability had not yet been ubiquitous during everyday life, and secondly during a seizure, parents tended to be agitated and it would have been asking too much from them to apply the carbogen bottle and at the same time video record their child. Equally a continuous video monitoring of the children during sleep would have been technically not feasible.

[3] You need to add to the manuscript why the 5% carbon dioxide and 95% oxygen are used. What were your reasons to avoid 7%/93% or other proportions?

**Response:** In the experiments leading to our medicinal product (the 6 liter carbogen bottle with attached mask) we have performed time and concentration serial experiments to achieve an inspiratory pCO_2_ partial pressure of 5 mmHg. This was the partial pressure that was efficient in the animal experiments and in the single incidences where carbogen had been successfully administered in single cases under EEG-monitoring.

[4] When the low-pressure can was used, what precautions were taken to avoid rebreathing? The reason that I asked this is that even if the carbogen composition is 5% CO2 and 95% O2, the expiratory air has 4% CO2 and 16% O2 composition, which can affect the results of your study.

**Response:** The design of the mask (loose fitting, small covered volume) and the continuous stream of fresh carbogen gas from the bottle prevented any rebreathing effect, which we had tested in our preliminary experiments in the run up to the study.

[5] What the caregivers were advised, they might forget, so giving written advice will be beneficial. Was written instruction given for the caregivers?

**Response:** We had provided a written and illustrated instruction to the parents how to use the bottle. This had also been translated in various languages to make sure that the parents understood the handling of the bottle.

[6] How can we reliably say that the abnormal body movement the child has at home is a febrile seizure? What if the child is having shivering, and the parents think it is a seizure? What if the child is not having a fever at the time of the seizure that occurred at home? What if the child is having a central nervous system infection?

**Response:** As mentioned in the paper, there was of course always a certain margin of uncertainty because we had to rely on patient reported outcomes. However, this uncertainty cannot be completely removed as it was in the nature of the study. As the parents had already experienced a seizure in their children before they were recruited to the study We thus assumed that the parents would be knowledgeable, how a seizure would look like in their child. To make sure that it was a febrile seizure, parents were instructed to measure the body temperature and report the fact that a seizure had occurred and the body temperature at the time of the seizure to the study team.

[7] What if the child has seizures and the family didn't report? How do you make sure that the families report and apply the carbogen or 100% oxygen whenever their child has a febrile seizure?

**Response:** As mentioned above, we were dependent on the parents to report any incidence where the child had a presumed seizure or when the study medication was used. Indeed, there have been incidents where seizures occurred and the parents did not apply the study medication (e.g. because they were shopping and had forgotten to take the bottle with them). These incidents were recorded in the patient case files, reported in the CONSORT flow sheet, but not included into the final efficacy analysis.

[8] How is the safety profile of the equipment? Is there a possibility that the equipment is malfunctioning and stopping air flow, which can potentially lead to rebreathing or suffocation?

**Response:** The bottles were tested and certified by the BfArM, which is the public entity in Germany responsible for the admission of medicinal products. As mentioned above, the mask was loose-fitting and the covered volume above the nose was small so that no rebreathing could occur. We have tested the bottle/mask combination in place without any gasflow and did not see a decline in oxygen saturation (measured by pulse oximetry) or an increase of transcutaneous pCO_2_ values.

[9] In line 106, you stated that the study was done between 2012 and 2015. What was the reason for not submitting the result and conclusion earlier?

**Response:** It was a negative finding and at the first place we had tried several pediatric journals, only to be rejected (despite the fact that those journals had also publicly stated to publish negative results). Then, honestly, we thought it would not be worth the while to pursue publishing our negative results. However, later we came to the conclusion that publication would be necessary, because the study had been registered in ClinicaTrials.gov and colleagues kept asking by email what had come out of the study.

[10] What was the reason for making the face mask loose-fitting?

**Response:** The mask had to be loose-fitting to prevent potential rebreathing and suffocation. The flow allowed to achieve 5 mmHg inspiratory pCO_2_ pressure over 3 minutes.

[11] The conclusion is not well suited for the results and the discussion of the study. A better conclusion would be: The study didn't show any superior benefit of carbogen over pure oxygen in ceasing febrile seizures within 3 minutes. There are possible different reasons that might mask the benefit of carbogen, such as the alkalosis reversal was not confirmed with blood gas analysis, diagnosing febrile seizure can be difficult for parents, and knowing when the seizure has stopped is difficult for the parents.

**Response:** We thank the reviewer for the suggestion and have amended our conclusion accordingly.

**Reviewer #3:**

Review to paper “The use of carbogen for interruption of febrile seizures - the randomized controlled CARDIF trial”

***Minor comments***

[1] In the abstract, on page 3, lines 43–44, it is not clear whether you are referring to the number of children or the number of febrile seizures: “The febrile seizure was terminated in 5/15 children on carbogen and in 8/11 children on oxygen.” I understood that the study medication was used in n = 30 febrile seizures (15 episodes in children within the verum arm, 11 episodes in children within the placebo arm, and 4 episodes in children within the verum open arm), in a total of 20 children. Check whether this could be clarified.

**Response:** We rephrased this sentence as follows: “The febrile seizure was terminated in 5/15 episodes on *carbogen* and in 8/11 episodes on *oxygen* (Fischers' exact test; p = 0.11).”

[2] On page 6, line 113, the sentence could be improved because it contains two actions (“have been published” and “are added”) that are not connected by a linking word such as “and” or “which.”

**Response:** “and” has been added.

[3] In Table 1, page 9, line 233, the number of patients in the verum arm indicates that 32 [69.6%] have one FS in history and 15 [32.6%] have more than one FS in history, which does not add up to 46. Verify if this is correct, since cases per gender indeed add up to 46.

**Response:** Thank you for pointing out this mistake. It should be 14 [30.4%] patients with more than one FS in history. This has now been corrected in Table 1.

[4] It would be stronger and clearer if, in the Methods section corresponding to “Randomisation and allocation,” the final number of patients randomised and followed, as well as those with recurrent febrile seizures, were stated. Additionally, it would be beneficial to refer to the CONSORT flowchart provided.

**Response:** The reference to the CONSORT flowchart has now been added. The chart has been amended and now provides the above asked information. Additionally, the number of patients with simple and complex febrile seizures in the treatment and placebo arms are now mentioned separately and discussed (see answer to reviewer #2).

[5] The section on power analysis, in my opinion, should not be part of the statistical analysis since it is mostly related to sample size determination, either as part of the explanation on how a desired sample size was decided or to contextualize the limitations faced during the interim phase of the clinical trial. It is not an actual analysis of the data obtained. An easy solution would be to put it as an individual subsection within the Methods.

**Response:** We agree and have now moved the "Power analysis" section into the Methods section / Study design.

[6] What is the reason for carrying out a conventional chi-squared test instead of a Fisher’s exact test? You have a small sample size; usually, Fisher’s exact test works better under such conditions (small sample size or when any expected cell count is <5). However, looking at your results, it would not make much of a difference. However, since you actually followed that kind of reasoning when you applied the Mann–Whitney U test, you should consider this.

**Response:** We agree with the reviewer and have now used the Fischer's exact test for all respective calculations.

[7] When comparing the patients in the crossover part of the study (6 patients in each group), the reasoning behind using a statistical test (regardless of whether it is a chi-squared test or a Mann–Whitney U test) is even more confusing. In my opinion, it is not worth relying on a formal null-hypothesis test with n = 6 vs. 6. You can run one, but the result will be practically uninterpretable and may mislead readers. It would be better to treat the data as descriptive/exploratory and focus on effect sizes, exact confidence intervals, and raw data. Formal testing is not useful here since the expected power is extremely low for any but enormous effects, so a nonsignificant p-value mostly reflects small n, not “no effect.” You already followed this logic in Table 2.

**Response:** We agree with the reviewer and have now only described the outcomes of the cross-over arm by mentioning the median and interquartile range.

[8] You include in your paper the CONSORT flowchart for participation; however, in no other place in the paper do you mention adherence to the CONSORT guidelines. If you adhered to any reporting guidelines, and also to guidelines for designing the trial (or good practice guidelines), you should mention it.

**Response:** The following sentence has been added to the methods section: The study was conducted according to the guidelines of Consolidated Standards of Reporting Trials (CONSORT) and good clinical practice. The filled in CONSORT questionnaire is now attached to the supplementary materials.

***Major comments***

[9] In relation to how the hypothesis is declared: I have some issues with the way the hypothesis is stated (page 6, lines 117–119), especially from a reader’s standpoint before reading the rest of the Methods section. You stated: “…Six liters of carbogen gas administered over a period of 3 minutes through a low-pressure can with an attached loose-fitting face mask is safe and more effective than placebo (100% oxygen) in interrupting recurrences of febrile seizures…” I assumed, based on what you explained throughout the paper, that the intention is to communicate that you want to assess whether the intervention stops the seizure during the 3-minute window while receiving the intervention. However, in my opinion, readers could understand that the intention is to communicate that the intervention’s goal is to prevent another seizure episode after receiving the intervention (3 minutes’ exposure to carbogen). The wording “interrupting recurrence” may imply this. If you say that the outcome of interest is “interrupting recurrences of febrile seizures” to a clinician, it would usually be understood as preventing another seizure from happening after the episode under treatment. For the reader, this could mean both stopping the ongoing seizure and giving a treatment that reduces the likelihood of subsequent seizures. I am sure your intention is to communicate as the outcome “stopping of an ongoing seizure” rather than preventing a new one; in that case, you are measuring termination. I think readers would benefit from a clearer statement of the hypothesis, similar to how the aim is stated: to determine whether 6 liters of carbogen gas (5% carbon dioxide + 95% oxygen) delivered from a low-pressure can would suppress acute febrile seizures (page X, lines 102–104).

**Response:** We agree that the wording might be easily misunderstood. As we only recruited children with at least one previous febrile seizure, any following seizure would be considered a "recurrence". The aim of the study was clearly to interrupt the ongoing seizure within the 3 minutes of carbogen application. We have changed the text to make this clear.

[10] In relation to the power analysis: If, in the most conservative scenario, you expected a power of 86% for a difference of 50% (75% vs. 25%), then when, in the preliminary phase, you obtained 15 episodes in one arm and 11 in the other, that would indeed impact power. This should be addressed either in the power analysis section (my recommendation) or somewhere in the Results (as a limitation within the interim results).

**Response:** We have added a sentence to the power analysis section that an unequal distribution of seizure events between treatment arms, which might occur in studies with a low number of patients / treatment events might impact the power analysis and increase the α (type I) error.

[11] In relation to reason for not observing the expected effect: In the Discussion, you are right to discuss the reasons why you did not observe a similar effect as previously reported by small hospital-based studies or animal studies, and I agree with you that difficulties in the administration of carbogen are a major determinant of not achieving proper exposure under such a critical situation. However, you should briefly discuss the possibility that carbogen (with 5% CO₂) is not actually effective in children. Is there any reason you may think this could be possible?

**Response:** Children with febrile seizures clearly have a higher frequency of increased blood pH values (alkalosis) due to hyperventilation as shown by Schuchmann et al. 2011 (PMID 21910730, Figure 1A). Using added CO_2_ in the inhaled air, would normalize the pH, which is basic respiratory physiology and would not differ between children and adults. The theory here is that children within the critical age group would react to their increased body temperature with an increased respiratory drive that depletes them of CO2 (respiratory alkalosis), which we strive to normalize by the inhaled CO2.
